# Chromone-Derived Polyketides from the Deep-Sea Fungus *Diaporthe phaseolorum* FS431

**DOI:** 10.3390/md17030182

**Published:** 2019-03-20

**Authors:** Heng Guo, Zhao-Ming Liu, Yu-Chan Chen, Hai-Bo Tan, Sai-Ni Li, Hao-Hua Li, Xiao-Xia Gao, Hong-Xin Liu, Wei-Min Zhang

**Affiliations:** 1 State Key Laboratory of Applied Microbiology Southern China, Guangdong Provincial Key Laboratory of Microbial Culture Collection and Application, Guangdong Open Laboratory of Applied Microbiology, Guangdong Institute of Microbiology, Guangzhou 510070, China; hengeguo163@163.com (H.G.); liuzm@gdim.cn (Z.-M.L.); chenyc@gdim.cn (Y.-C.C.); maibao66@126.com (S.-N.L.); lihh@gdim.cn (H.-H.L.); 2 College of Pharmacy, Guangdong Pharmaceutical University, Guangzhou 510006, China; 3 Program for Natural Products Chemical Biology, Key Laboratory of Plant Resources Conservation and Sustainable Utilization, Guangdong Provincial Key Laboratory of Applied Botany, South China Botanical Garden, Chinese Academy of Sciences, Guangzhou 510650, China; tanhaibo@scbg.ac.cn

**Keywords:** *Diaporthe phaseolorum*, deep-sea derived fungus, chromone-derived polyketides, phaseolorins A-F

## Abstract

Five new chromone-derived polyketides phaseolorins A-F (**1**–**5**), together with nine known compounds, were isolated from the deep-sea derived fungus *Diaporthe phaseolorum* FS431. The structures of new compounds were determined by analysis of their NMR and high-resolution electrospray ionization mass spectroscopy (HRESIMS) spectroscopic data. The absolute configurations were confirmed by chemical transformations, extensively experimental electron capture detection (ECD) calculations, or X-ray crystallography. Among them, compound **2** represented the first example for a new family of chromone derivative possessing an unprecedented recombined five-member *γ*-lactone ring. Moreover, the new compounds (**1**–**5**) were evaluated for in vitro cytotoxic activities against a panel of human cancer cell lines.

## 1. Introduction

Marine-derived fungi are emerging as a promising source for discovering novel natural products with significant biological and pharmacological properties. Deep-sea fungi have attracted even more attention in recent years because they are naturally forced to metabolize more potent bioactive compounds to acclimatize the extreme and variable environments of the deep sea, which has resulted in the generation of numerous chemically diverse and structurally unique secondary metabolites. Therefore, deep-sea fungi have been proverbially respected to be one of the most potential resources for the discovery of biologically meaningful natural products [1,2].

**Figure 1:**
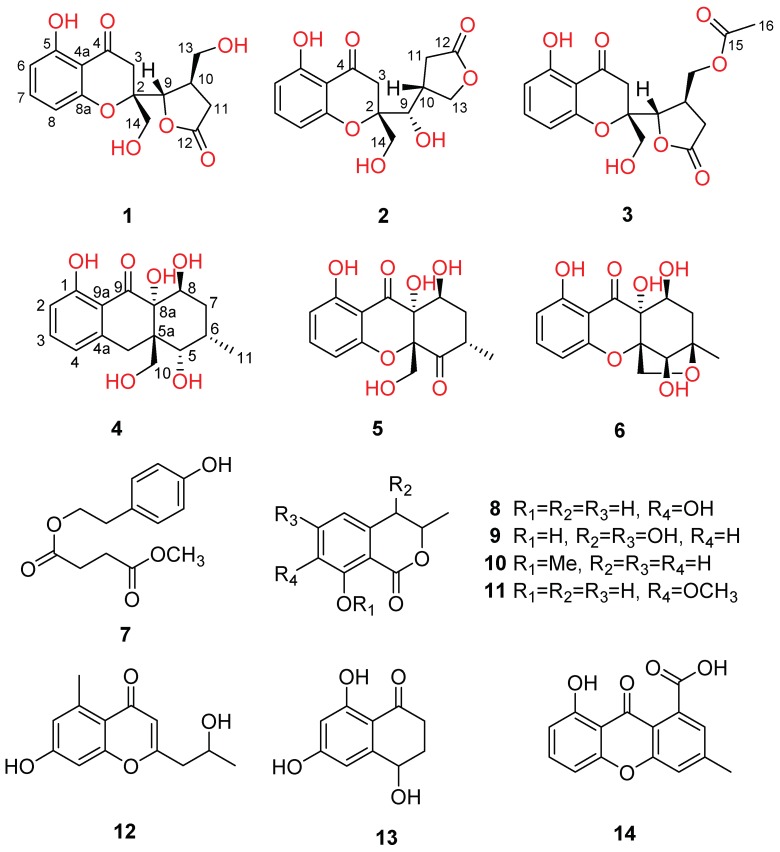
Chemical structures of compounds **1**–**14**.

Previous studies towards the fungi of the genus Diaporthe have revealed that these fungi are capable of producing structurally fascinating and architecturally diverse natural products, such as polyketides, highly substituted benzophenone, pyran-2-one, terpenoid, and diapolic acid [3,4,5,6,7], which usually showed potent cGMP-dependent protein kinase inhibitory and antiparasitic [4], antibacterial [8,9], antimalarial [10], and cytotoxic activities [11,12]. In the course of our continuing search for biologically meaningful and structurally unique compounds, the deep-sea fungus *Diaporthe phaseolorum* FS431, collected from the Indian Ocean (depth 3605 m, 7°57.75944′ N, 89°19.43851′ E), has attracted our attention, and the following phytochemical study resulted in the isolation of five new chromone-derived polyketides (phaseolorins **1**–**5**) (Figure 1) together with nine known derivatives identified as phomoxanthone G (**6**) [13], 4-hydroxyphenthyl methyl succinate (**7**) [14], 7,8-dihydroxy-3-methyl-3,4-dihydroisocoumarin (**8**) [15,16], 4,6-dihydroxymellein (**9**) [17], *O*-methylmellein (**10**) [18], 8-hydroxy-3-methyl-3,4-dihydroisocoumarins (**11**) [19], 5-carbomethoxymethyl-2-heptyl-7-hydroxychromone (**12**) [20], 6-hydroxyisosclerone (**13**) [21], and monodictyxanthone (8-hydroxy-3-methyl-9-oxo-9H-xanthene-1-carboxylic acid) (**14**) [22]. Herein, the details of isolation, structural elucidation by NMR spectral interpretation, X-ray diffraction, quantum chemistry calculation, and biological evaluation of these isolates are described.

## 2. Results and Discussion

### 2.1. Structure Elucidation

Compound **1** was obtained as a yellow powder. Its molecular formula was established as C_15_H_16_O_7_ on the basis of its high-resolution electrospray ionization mass spectroscopy (HRESIMS) with the protonated molecular peak discovered at *m*/*z* 309.0971 ([M + H]^+^, calcd 309.0969), requiring eight unsaturation degrees. The IR spectrum displayed the classical absorption bands at 3362, 1771, and 1614 cm^−1^, which were characteristic for the hydroxy, *γ*-lactone carbonyl, and conjugated ketone carbonyl functionalities, respectively. The ^1^H NMR spectrum of **1** exhibited a series of proton resonances responsive for two hydroxymethyls at *δ*_H_ 3.78 (m, H-14) and 3.59 (m, H-13) and three aromatic ones of a 1,2,3-trisubstituted benzene at *δ*_H_ 6.43 (d, *J* = 8.3 Hz, H-6 and H-8) and 7.38 (t, *J* = 8.3 Hz, H-7). The ^13^C NMR spectrum showed two typical carbon resonances at *δ*_C_ 179.0 and 198.1, which were unambiguously supported the presence of a *γ*-lactone and ketone carbonyl functional groups, respectively.

**Figure 2:**
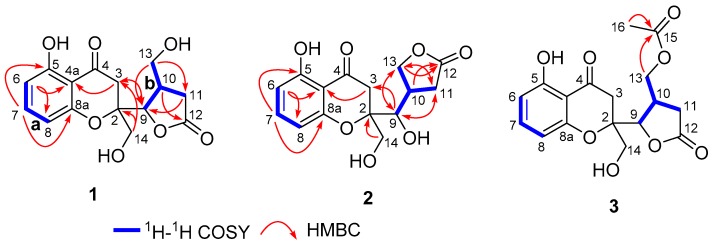
^1^H–^1^H correlated spectroscopy (COSY) and key HMBC correlations of **1**–**3**.

With a careful inspection and analyses of the ^13^C NMR data (Table 1) in conjunction with HSQC spectrum, it could readily reveal the existence of 15 carbon resonances including four methylenes, five methines, and six nonprotonated carbons. Notably, an elaborative comparison of its 1D (Table 1) and 2D NMR spectra data of **1** with those of the known compound mangorvamide H [23] disclosed that compound **1** should also share the similar chromone-derived skeleton. The major differences between **1** and mangorvamide H were attributed to the replacements of the C-13 and C-14 methyls in the known compound with hydroxymethyls in **1**, and the assumption could be rationally verified by the ^13^C NMR chemical shifts (*δ*_C_ 64.6 (C-13), 62.9 (C-14)) and the critical HMBC correlations from H_2_-14 (*δ*_H_ 3.78 (m)) to C-2 and C-3 as well as H_2_-13 (*δ*_H_ 3.59 (m)) to C-9, C-10, and C-11 (Figure 2).

**Figure 3:**
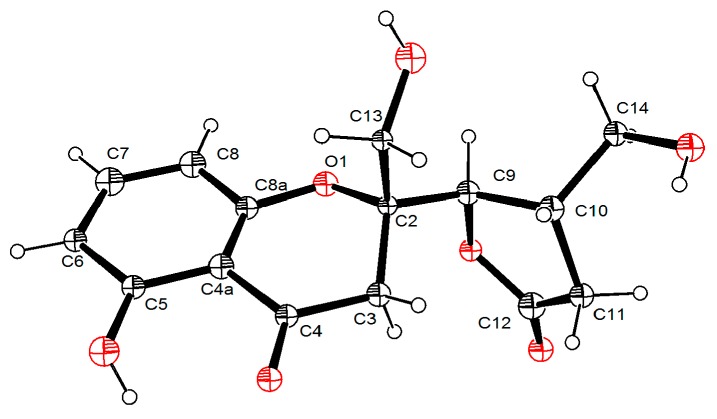
Perspective drawing of the X-ray structure of **1**.

**Figure 4:**
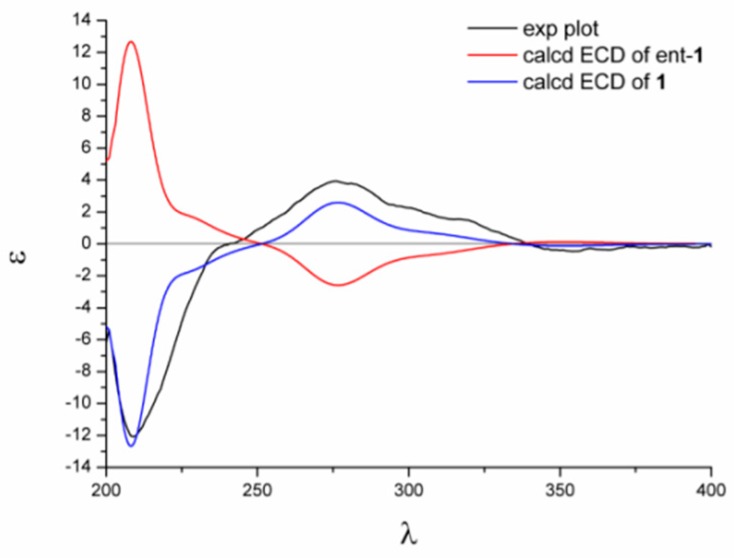
Experimental and calculated electron capture detection (ECD) spectra of **1**.

The relative configuration of compound **1** was established with the aid of the single-crystal X-ray diffraction experiment, which was successfully carried out using CuK*α* radiation (Figure 3). Although it unfortunately failed to clarify its absolute configuration, it unambiguously revealed the planar and relative configuration of compound **1** as depicted in Figure 1. The absolute configuration of **1** was finally determined by the calculated CD spectrum method, as expected, it provided a satisfying agreement between the calculated CD spectrum for the 2*S*,9*S*,10*R* isomer and the experimental one (Figure 4). Therefore, the structure elucidation of compound **1** was completely finished, and its absolute structure was deduced to be 2*S*,9*S*,10*R* and trivially named as phaseolorin A.

Compound **2** was also purified as yellow powder. It has the same molecular formula C_15_H_16_O_7_ as that of **1** based on the positive HRESIMS (*m*/*z* 331.0794 [M + Na]^+^; calcd for C_15_H_16_O_7_Na, 331.0788). Analysis of its ^1^H and ^13^C NMR data revealed similar structure features to those of **1**, which in turn gave rise to the conclusive information for the structural isomeric relationship between **1** and **2**. The further careful interpretation of the HMBC correlations could reconfirm this deduction. The major differences between **1** and **2** were the noticeable chemical shifts of C-9 (*δ*_C_ 84.4 in **1**; 73.1 in **2**) and C-13 (*δ*_C_ 64.6 in **1**; 71.5 in **2**) together with the critical HMBC correlation from H_2_-13 to C-12 (*δ*_C_ 177.1) in **2**, indicating that they might be a pair of regioisomers with the formation of a new five-member *γ*-lactone ring in compound **2**. Notably, compound **2** was discovered as the first natural product possessing such a five-member *γ*-lactone ring from nature.

Furthermore, the experimental electron capture detection (ECD) spectrum of **2** (Figure S18) was very similar to that of compound **1**, which showed good accordance with the theoretical one with obvious Cotton effects found at 353 nm. The aforementioned informative results strongly suggested that the absolute stereogenic centers of compound **2** might be the same as those of compound of **1** and be rationally clarified as 2*S*,9*S*,10*R*. Moreover, the compounds **1** and **2** should share the same biosynthetic pathway, which could further strengthen this conclusion. In order to achieve the direct evidence to unambiguously determine the absolute configuration of compound **2**, the further chemical transformations between compounds **1** and **2** using the various chemical reactions were performed.

**Figure 5:**
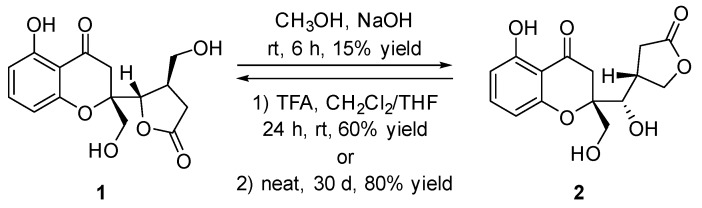
The mutual chemical transformation between compounds **1** and **2**.

After an extensive screen of chemical transformations in different base or acid conditions, the compound **2** was disclosed to be readily transformed to compound **1** under the trifluoroacetic acid (TFA) condition with the CH_2_Cl_2_/THF (3:1) as the combined solvent (60% yield, 24 h) or under the neat state in air condition at room temperature (80% yield, 30 d); whereas the compound **2** could be correspondingly transformed into compound **1** under a methanol solution of 0.25 M NaOH as the strong based condition (15% yield, 6 h) (Figure 5). The characteristic for the mutual transformation between compounds **1** and **2** thus successfully established that the absolute configuration of compound **2** was the same with that of compound **1** and assigned as 2*S*,9*S*,10*R*. Therefore, the absolute structure of compound **2** was completely established, and it represented a new family of chromone derivatives and was given the trial name as phaseolorin B.

**Figure 6:**
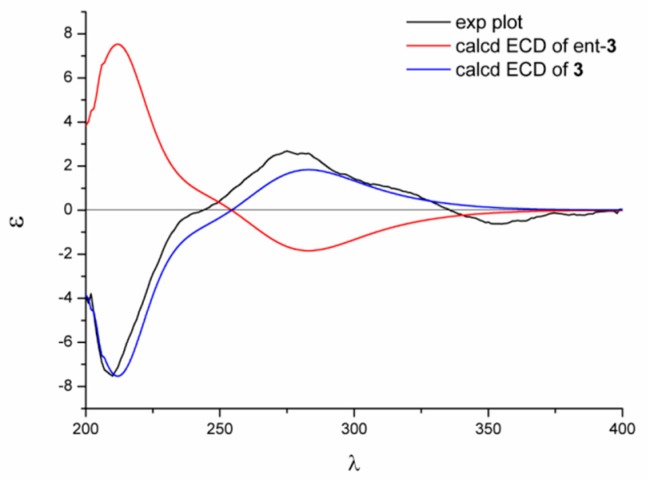
Experimental and calculated ECD spectra of **3**.

Compound **3** was isolated as yellow oil. The molecular formula was absolutely assigned as C_17_H_18_O_8_, attributable to the positive ion mode HRESIMS (*m*/*z* 351.1072 [M + H]^+^ (calcd for C_17_H_19_O_8_, 351.1074), corresponding to nine degrees of unsaturation. The ^1^H and ^13^C NMR spectra of **3** closely resembled to those of **1** and **2**, collectively pointing to the reality of a chromone-derived derivative for compound **3** as those for compounds **1** and **2**. The major differences between the compounds **3** and **1** were attributed to the existence of two additional carbons (*δ*_C_ 171.0, 20.7) in **3** (Table 2), which were characteristically responsive for an acetyl group. As referring to the 2D NMR spectra, the key HMBC correlations from H_3_-16 to C-15 and H_2_-14 to C-15 could be obviously distinguished, indicating the acetoxy ought to be located at C-14. The absolute configuration of **3** was theoretically deduced to be the same as that of **1** with the aid of the calculated CD spectrum method, which expectedly launched a calculated CD spectrum of the 2*S*,9*S*,10*R* isomer perfectly matched with the experimental one (Figure 6). Therefore, the configuration of **3** was conclusively assigned as shown in Figure 1 and given the tentative name as phaseolorin C.

**Figure 7:**
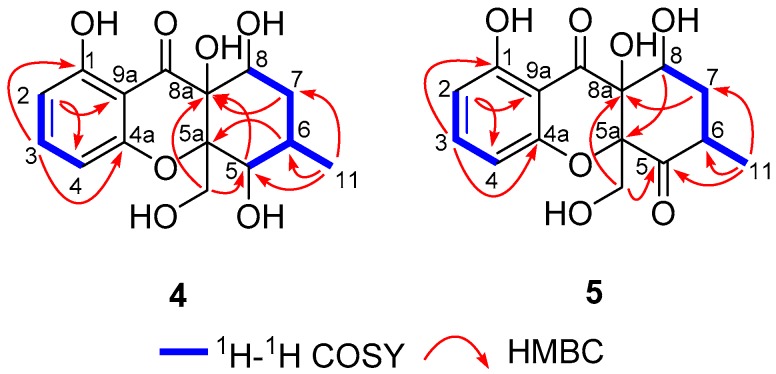
^1^H–^1^H COSY and key HMBC correlations of **4** and **5**.

Compound **4** was obtained as yellow crystals. Its molecular formula was assigned as C_15_H_18_O_7_ because of the positive mode HRESIMS with a protonated molecular peak observed at *m*/*z* 311.1124 ([M + H]^+^, calcd 311.1125), which clearly suggested the presence of seven indices of unsaturation. The ^1^H NMR spectrum of **4** showed a series of characteristic signals for a 1,2,3-trisubstituted benzene ring [*δ*_H_ 6.46 (dd, *J* = 8.3, 0.9 Hz), 6.57 (dd, *J* = 8.3, 0.9 Hz), 7.41, (t, *J* = 8.3 Hz)] and a methyl group (*δ*_H_ 1.11 (d, *J* = 6.7 Hz)). Moreover, the ^13^C NMR data (Table 3) coupling with the HSQC spectrum of **1** further successfully clarified the existence of 15 carbon resonances corresponding to one methyl, two methylenes, six methines, and six quaternary carbons including a carbonyl one. Furthermore, the ^1^H−^1^H correlated spectroscopy (COSY) and HSQC spectra unambiguously disclosed the presence of two spin coupling systems as depicted with bold lines in Figure 7: **a** (H-1/H-2/H-3) and **b** (H-5/H-6/H-7/H-8, H-6/H-11). These typical NMR characteristics strongly suggested that compound **4** should share a classic chromone scaffold.

After a careful inspection of the NMR spectra of **4** with those of the known chromone mangrovamide J [23], it could be readily disclosed that they showed very close similarity in all the NMR spectra with most profiles. The major differences between them were the methyl moiety at C-5a in the known compound mangrovamide J was replaced by a hydroxymethyl one in **4** and the absence of a double bond at C-6 and C-7 positions in **4**, which were substantiated by its chemical shifts (*δ*_H_ 4.38; *δ*_C_ 68.5) in conjunction with the HMBC correlations from H_2_-10 (*δ*_H_ 4.38, (t, *J* = 2.9 Hz)) to C-5, C-5a, and C-8a as well as H_3_-11 to C-5, C-6, and C-7. Therefore, the planar structure of **4** was elucidated as shown in Figure 1.

**Figure 8:**
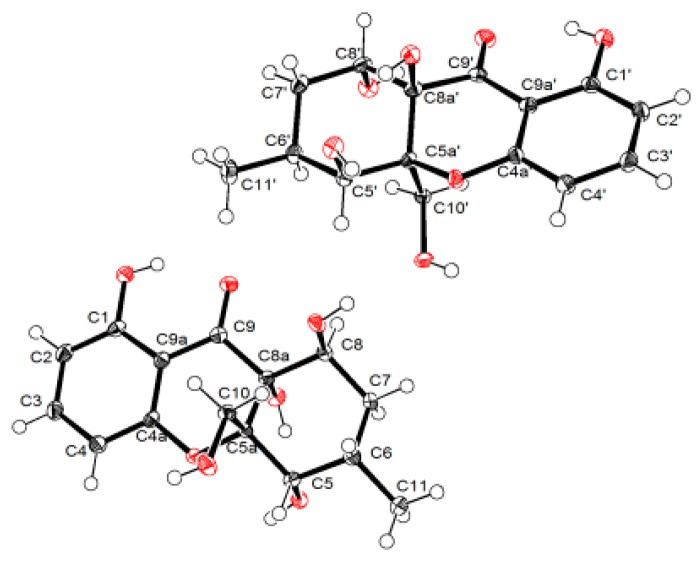
Perspective drawing of the X-ray structure of **4**.

With the aim to grow its single crystals, many tentative efforts by using different solvents or solvent combinations were conducted. Fortunately, a single crystal of **4** was successfully obtained from the methanol solution after repeated attempts. The following single-crystal X-ray diffraction experiment was carried out using CuK*α* radiation with a Flack parameter of 0.00(4) (Figure 8), which unambiguously verified the absolute configuration of compound **4**. Thus, the structure elucidation of compound **4** was completely finished, and its absolute structure was finally deduced to be 5*S*,5a *S*,8*S*,8a*R* and trivially named as phaseolorin D.

Compound **5** was obtained as yellow oil. Its molecular formula was established as C_15_H_16_O_7_ on the basis of the protonated molecule peak at *m*/*z* 331.0788 [M + Na]^+^ in its HRESIMS, requiring eight degrees of unsaturation. The 1D NMR data (Table 3) of **5** were almost in accordance with those of **4**, except for the possible replacement of hydroxyl group at C-5 position in **4** by an additional carbonyl functionality (*δ*_C_ 207.1) in **5**, which could be further strengthened by the determinative ^1^H-^1^H COSY cross-peaks of H-6/H-7/H-8 and the predominant HMBC correlations from H_3_-11 to C-5, C-6, and C-7, as well as H_2_-7 to C-5. Moreover, this deduction could be also evidenced by the extensive 2D NMR analysis as depicted in Figure 2. Interestingly, compound **5** showed an ECD spectrum almost consistent with that of **4** (see supporting information), which strongly illustrated a mode of sharing a very similar absolute configuration by the consideration of the same biogenesis. Therefore, the configuration of **5** was conclusively assigned as shown in Figure 1 and given the trial name as phaseolorin E.

### 2.2. Biological Activity

Compounds **1**–**6** were evaluated for cytotoxicities against three human cancer cell lines: HepG-2 (liver cancer), MCF-7 (breast cancer), and SF-268 (human glioblastoma carcinoma). However, all of them were found to be devoid of significant cytotoxicity activity even at a concentration of 100 µM.

## 3. Materials and Methods

### 3.1. General Experimental Procedures

Optical rotations were obtained on an Anton Paar MCP-500 spectropolarimeter (Anton Paar, Graz, Austria) with MeOH as solvent at 20 °C. UV spectra were recorded on a Shimadzu UV-2600 spectrophotometer (Shimadzu, Kyoto, Japan). IR data were acquired on a Shimadzu IR Affinity-1 spectrometer (Shimadzu, Kyoto, Japan). CD spectra were determined using a Jasco 820 spectropolarimeter (Jasco Corporation, Kyoto, Japan). 1D and 2D NMR spectra were obtained on a Bruker Avance 500 MHz or 600 MHz NMR spectrometer (Bruker, Fällanden, Switzerland) using TMS as an internal standard. High-resolution electrospray ionization mass spectroscopy (HRESIMS) and electrospray ionization-mass spectrometry (ESIMS) data were measured respectively on a Thermo MAT95XP high resolution mass spectrometer (Thermo Fisher Scientific, Bremen, Germany) and an Agilent Technologies 1290-6430A Triple Quad LC/MS (Agilent Technologies, Palo Alto, CA, USA). Preparative HPLC collection used a C_18_ column (YMC-pack ODS-A, 250 × 20 mm, 5 μm, 12 nm, YMC Co., Ltd., Kyoto, Japan). Semipreparative HPLC separations were performed utilizing a C_18_ column (YMC-pack ODS-A/AQ, 250 × 10 mm, 5 μm, 12 nm, YMC CO., Ltd., Kyoto, Japan). Column chromatography (CC) was performed on silica gel (200−300 mesh, Qingdao Marine Chemical Inc., Qingdao, China) and Sephadex LH-20 (Amersham Biosciences, Uppsala, Sweden). Solvents for isolation were analytical grade.

### 3.2. Fungal Material

The fungus strain FS431 was isolated from a marine sediment sample collected from the Indian Ocean (depth 3605m, 7°57.75944′ N/ 89°19.43851′ E) in March 2016 and identified as *Diaporthe phaseolorum* based on sequencing of the internal transcribed spacer (ITS) region (Accession No. MK459544) with 99% similarity to *Diaporthe phaseolorum* MJ14 (Accession No. KM203581). The strain was deposited at the Guangdong Provincial Key Laboratory of Microbial Culture Collection and Application, Guangdong Institute of Microbiology.

### 3.3. Fermentation and Extraction

*Diaporthe phaseolorum* FS431 was cultured for 5 days at 28 °C in a potato dextrose agar (PDA) culture plate. The mycelial plugs were transferred to ten 500-mL Erlenmeyer flasks each containing 250 mL potato dextrose broth (20% potato, 2% glucose, 0.3% KH_2_PO_4_, 0.15% MgSO_4_•7H_2_O, and 250 mL water with 1.5% sea salt), and then incubated on a rotary shaker at 120 r/m and 28 °C for 4 days as seed cultures. After that, each of the seed cultures (10 mL) was transferred into autoclaved 1000-mL Erlenmeyer flasks with 500 mL potato dextrose broth. Then, the strain was incubated on a rotary shaker for 7 days at 28 °C and 120 r/m. The culture (120 L) was centrifuged to give the broth and mycelia. The broth was exhaustively extracted with EtOAc for four times, and then the EtOAc layers were combined and evaporated under reduced pressure at a temperature not exceeding 40 °C to yield a dark brown gum (53 g).

### 3.4. Isolation and Purification

The crude extract was fractionated by a silica gel column eluting with step gradient petroleum ether/EtOAc (*v*/*v* 30:1, 20:1, 10:1, 7:1, 9:2, 3:1, 2:1, 1:1, 0:1) and CH_2_Cl_2_/MeOH (*v*/*v* 5:1, 1:1, 0:1) to obtain 20 fractions (Fr.1-Fr.20) based on thin-layer chromatography (TLC) analysis. Fr.13 was chromatographed over ODS using gradient elution of H_2_O-MeOH (30–100%) to get three subfractions Fr.13-1-Fr.13-3. Subfraction Fr.13-3 (121.8 mg) was rechromatographed over silica gel (*n*-hexane/EtOAc, 5:1, 2:1, 1:1; CH_2_Cl_2_/MeOH, 10:1, *v*/*v*) followed by semipreparative HPLC separation (MeOH-H_2_O, 45:55, 3 mL/min) to afford compound **4** (4.9 mg, *t*_R_ = 20.5 min).

Fr.14 (3.2 g) was further separated on a Sephadex LH-20 column with MeOH to provide seven subfractions (Fr.14-1-Fr.14-7). Fr.14-2 (251.7 mg) was separated by preparative HPLC eluting with MeOH-H_2_O (46:54, 3 mL/min) to give four subfractions (Fr.14-2-1-Fr.14-2-4). Subfraction Fr.14-2-1 (124.9 mg) was separated by silica gel (CH_2_Cl_2_/MeOH, 100:1, 50:1, 30:1, 20:1, 10:1, *v*/*v*), and finally purified by semipreparative HPLC (MeOH-H_2_O, 30:70, 3 mL/min) to obtain compound **5** (2.5 mg, *t*_R_ = 13.3 min).

Fr.16 (1.8 g) was separated by CC over Sephadex LH-20 (CH_2_Cl_2_/MeOH, 1:1) to give six subfractions (Fr.16-1-Fr.16-6). Fr.16-3 (266.5 mg) was purified by CC over silica gel with the mobile phase of *n*-hexane/EtOAc (10:1, 8:1, 5:1, 2:1, 1:1), following by a semipreparative HPLC (MeOH-H_2_O, 50:50, 3 mL/min) to afford compound **3** (2.3 mg, t_R_ = 28.7 min). Compounds **1** (13 mg, *t*_R_ = 10.0 min) and **2** (5.5 mg, *t*_R_ = 15.0 min) were obtained from Fr.16-4 (161.4 mg) by silica gel CC (CH_2_Cl_2_/MeOH, 50:1, 30:1, 20:1, 10:1, *v*/*v*) with a final purification on a chiral-phase column (DAICEL IC, 2-propanol/*n*-hexane, 50:50, 3 mL/min).

Phaseolorin A (**1**): yellow powder; ${\left[\alpha \right]}_{D}^{20}$ +36.5 (*c* 0.1, MeOH); UV (MeOH) *λ*_max_ (log *ε*) 211 (6.70), 273 (6.11), 348 (5.70) nm; CD Δε (0.18 mg/mL, MeOH) *λ*_max_ (Δ*ε*) 276 (+0.14), 354 (−1.19) nm; IR *ν*_max_ 3362, 2920, 1771, 1614, 1456, 1373, 1219, 1016, cm^−1^; ^1^H and ^13^C NMR data, see Table 1; (+)-HRESIMS *m*/*z* 309.0971 [M + H]^+^ (calcd for C_15_H_17_O_7_, 309.0969).

Phaseolorin B (**2**): yellow powder; $\;{\left[\alpha \right]}_{D}^{20}$ − 33.6 (*c* 0.1, MeOH); UV (MeOH) *λ*_max_ (log *ε*) 206 (6.35), 273 (5.98), 348 (5.55) nm; CD Δε (0.18 mg/mL, MeOH) *λ*_max_ (Δ*ε*) 281 (+0.81), 353 (−0.48) nm; IR *ν*_max_ 3399, 2918, 1771, 1624, 1458, 1361, 1223, 1053, cm^−1^; ^1^H and ^13^C NMR data, see Table 1; (+)-HRESIMS *m*/*z* 331.0794 [M + Na]^+^ (calcd for C_15_H_16_O_7_Na, 331.0788).

Phaseolorin C (**3**): yellow oil; $\;{\left[\alpha \right]}_{D}^{20}$ + 28.9 (*c* 0.1, MeOH); UV (MeOH) *λ*_max_ (log *ε*) 207 (6.33), 273 (5.98), 349 (5.55) nm; CD Δε (0.20 mg/mL, MeOH) *λ*_max_ (Δ*ε*) 275 (+1.42), 362 (−0.26) nm; IR *ν*_max_ 3358, 2918, 2849, 1770, 1732, 1645, 1625, 1578, 1462, 1362, 1225, 1016 cm^−1^; ^1^H and ^13^C NMR data, see Table 2; (+)-HRESIMS *m*/*z* 351.1072 [M + H]^+^ (calcd for C_17_H_19_O_8_, 351.1074).

Phaseolorin D (**4**): yellow crystals; $\;{\left[\alpha \right]}_{D}^{20}$ + 74.6 (*c* 0.1, MeOH); UV (MeOH) *λ*_max_ (log *ε*) 209 (6.05), 278 (5.75), 354 (5.28) nm; CD Δε (0.20 mg/mL, MeOH) *λ*_max_ (Δ*ε*) 279 (+6.85), 326 (+3.64), 293 (−0.79) nm; IR *ν*_max_ 3335, 2933, 1651, 1626, 1462, 1223, 1020, cm^−1^; ^1^H and ^13^C NMR data, see Table 3; (+)-HRESIMS *m*/*z* [M + H]^+^ *m*/*z* 311.1124, (calcd for C_15_H_18_O_7_, calcd 311.1125.

Phaseolorin E (**5**): yellow oil; $\;{\left[\alpha \right]}_{D}^{20}$ + 119.4 (*c* 0.1, MeOH); UV (MeOH) *λ*_max_ (log *ε*) 210 (6.91), 277 (6.14), 354 (5.28) nm; CD Δε (0.19 mg/mL, MeOH) *λ*_max_ (Δ*ε*) 289 (+4.85), 331 (+1.47), 358 (−0.51) nm; IR *ν*_max_ 3366, 2926, 2853, 1732, 1645, 1626, 1462, 1219, 1059, 1026 cm^−1^; ^1^H and ^13^C NMR data, see Table 3; (+)-HRESIMS *m*/*z* 331.0788 [M + Na]^+^ (calcd for C_15_H_16_O_7_Na, 331.0788).

### 3.5. X-ray Crytallographic Data of Compounds ***1*** and ***4***

The single-crystal X-ray diffraction data for compounds (**1** and **4**) were collected at 100K on Agilent Xcalibur Nova single-crystal diffractometer using CuK*α* radiation. Their crystal structures were refined by the program with full-matrix least-squares calculation. Hydrogen atoms bonded to carbons were located by the geometrically ideal positions by the “ride on” method. Hydrogen atoms bonded to oxygen were placed on the difference Fourier method and were included in the calculation of structure factors with isotropic temperature factors. Crystallographic data for the reported structures have been deposited in the Cambridge Crystallographic Data Centre. (Deposition number: CCDC 1890682 for **1**, CCDC 1890683 for **4**). Copies of the data can be obtained, free of charge, on application to the CCDC, 12 Union Road, Cambridge CB2 1EZ, UK (fax, +44-(0)-1223-336033; e-mail, deposit@ccdc.cam.ac.uk).

### 3.6. The Chemical Transformation Between Compounds ***1*** and ***2***

Sodium hydroxide (10 mg, 0.25 mmol) was slowly dissolved in anhydrous methanol (1 mL) at room temperature. To this clear solution, compound **1** (3.0 mg) was added carefully and the mixture stirred for 6 h under air condition. The crude mixture was then quenched with 2 N HCl (2 mL) and extracted with ethyl acetate (3 × 5 mL), washed with brine, and concentrated in vacuum. The crude product was purified by flash chromatography (silica gel, CHCl_3_/MeOH, 20:1→10:1) to provide **2** (0.45 mg, 15% yield) as a slight yellow powder.

A flame-dried 5 mL flask containing a mixture solvent of THF (0.25 mL) and CH_2_Cl_2_ (0.75 mL) was charged with compound **2** (1.5 mg) and TFA (20 μL), and the reaction mixture stirred vigorously under air condition at room temperature for 24 h. The mixture was quenched by addition of 2 mL water and extracted with ethyl acetate (5 × 3 mL). The combined organic phases were washed with brine (4 mL), dried over Na_2_SO_4_, and filtered. Removal of solvent by rotary evaporation and purification by flash column chromatography (silica gel, CHCl_3_/MeOH, 20:1→10:1) afforded **1** (0.90 mg, 60% yield).

The compound **2** (2.5 mg) was put at a 5 mL round flask and kept in neat state under air condition at the room temperature for ~30 d. Then, the resulting mixture was purified by 5 cm long flash chromatography (silica gel, CHCl_3_/MeOH, 20:1→10:1) afforded the desired product **1** (2.0 mg) with 80% yield.

### 3.7. Cytotoxic Activity Assay

The cytotoxic activities of compounds (**1**–**6**) against HepG-2, MCF-7 and SF-268 cell lines were evaluated by using the Sulforhodamine B (SRB) method [24] with cisplatin as the positive control.

### 3.8. Details of ECD Calculations

Merck molecular force field (MMFF) and DFT/TD-DFT calculations were carried out with the Spartan’14 software (Wavefunction Inc., Irvine, CA, USA) and the Gaussian 09 program, respectively [25]. Conformers within the 10 kcal mol^−1^ energy window were generated and optimized using DFT calculations at the b3lyp/6-31+g(d,p) level. Frequency calculations were performed at the same level to confirm that each optimized conformer was true minimum and to estimate their relative thermal free energy (ΔG) at 298.15 K. Conformers with the Boltzmann distribution over 5% were chosen for ECD calculations in methanol at the b3lyp/6-311+g(d,p) level. Solvent effects were taken into consideration using the self-consistent reaction field (SCRF) method with the polarizable continuum model (PCM) [26]. The ECD spectrum was generated by the SpecDis program [27] using a Gaussian band shape with 0.26 eV exponential half-width from dipole-length dipolar and rotational strengths.

## 4. Conclusions

The chemical investigation of the deep-sea-derived fungus *D. phaseolorum* FS431 has led to five new chromone-derived polyketides, named phaseolorin A–E (**1**–**5**), together with nine known compounds (**6**–**14**). Their structures were elucidated by the detailed analysis of spectroscopic data and single-crystal X-ray diffraction. However, none of compounds showed cytotoxic effects against HepG-2, MCF-7, and SF-268 cell lines.

## Figures and Tables

**Table 1:** ^1^H (600 MHz) and ^13^C (150 MHz) NMR spectroscopic data of **1** and **2**.

No.	1 ^a^		2 ^b^	
	*δ*_H_ (*J* in Hz)	*δ*_C_	*δ*_H_ (*J* in Hz)	*δ*_C_
2		84.9, C		86.4, C
3*α*	3.15, d, (17.4)	38.4, CH_2_	3.15, d, (17.4)	39.4, CH_2_
3*β*	3.03, d, (17.4)		3.05, d, (17.4)	
4		198.1, C		199.1, C
4a		108.3, C		109.2, C
5		162.7, C		162.4, C
6	6.43, d, (8.3)	108.6, CH	6.42, d, (8.3)	108.2, CH
7	7.38, t, (8.3)	139.6, CH	7.40, t, (8.3)	139.0, CH
8	6.43, d, (8.3)	110.1, CH	6.40, d, (8.3)	108.2, CH
8a		160.7, C		160.7, C
9	4.65, d, (2.9)	84.4, CH	4.04, t, (5.3)	73.1, CH
10	2.93, m	38.3, CH	3.15, m	38.2, CH
11*α*	2.83, dd, (18.2, 10.1)	32.0, CH_2_	2.66, dd, (17.3, 10.2)	30.1, CH_2_
11*β*	2.38, dd, (18.2, 3.1)		2.50, dd (17.3, 8.7)	
12		179.0, C		177.1, C
13*α*	3.59, m	64.6, CH_2_	4.51, t, (9.0)	71.5, CH_2_
13*β*			4.08, t, (9.0)	
14	3.78, m	62.9, CH_2_	3.91, br s,	63.4, CH_2_

^a^ Recorded in methanol-*d*_4_; ^b^ Recorded in acetone-*d*_6_.

**Table 2:** ^1^H (600 MHz) and ^13^C (150 MHz) NMR spectroscopic data of **3** in CD_3_COCD_3_.

No.	3		No.	3	
	*δ*_H_ (*J* in Hz)	*δ*_C_		*δ*_H_ (*J* in Hz)	*δ*_C_
2		84.7, C	9	4.66, d, (3.2)	83.0, CH
3*α*	3.14, d, (17.4)	38.1, CH_2_	10	3.27, m	35.1, CH
3*β*	3.03, d, (17.4)		11*α*	2.89, dd, (18.2, 10.2)	31.7, CH_2_
4		197.8, C	11*β*	2.42, dd, (18.2, 3.7)	
4a		108.1, C	12		175.9, C
5		162.4, C	13	4.20, d, (6.0)	66.3, CH_2_
6	6.44, d, (8.3)	108.3, CH	14	3.89, d, (3.0)	63.0, CH_2_
7	7.41, t, (8.3)	139.2, CH	15		171.0, C
8	6.44, d, (8.3)	109.6, CH	16	2.02, s	20.7, CH_3_
8a		160.4, C			

**Table 3:** ^1^H and ^13^C NMR spectroscopic data of **4** and **5** in CD_3_OD.

No.	4 ^a^		5 ^b^	
	*δ*_H_ (*J* in Hz)	*δ*_C_	*δ*_H_ (*J* in Hz)	*δ*_C_
1		163.4, C		163.6, C
2	6.46, dd, (8.3, 0.9)	109.7, CH	6.51, dd, (8.3, 0.9)	110.4, CH
3	7.41, t, (8.3)	139.0, CH	7.46, t, (8.3)	139.6, CH
4	6.57, dd, (8.3, 0.9)	109.5, CH	6.65, dd, (8.3, 0.9)	109.9, CH
4a		160.3, C		159.0, C
5	4.25, m	74.5, CH		207.1, C
5a		76.4, C		93.6, C
6	2.22, m	29.2, CH	3.01, m	38.1, CH
7*α*	2.04, m	32.0, CH_2_	2.11, m	39.2, CH_2_
7*β*	1.57, m			
8	4.38, t, (2.9)	68.5, CH	4.51, t, (2.7)	67.0, CH
8a		85.4, C		79.2, C
9		196.2, C		196.2, C
9a		108.6, C		107.9, C
10*α*	4.24, d, (13.5)	60.5, CH_2_	3.86, d, (13.2)	62.5, CH_2_
10*β*	3.77, d, (13.5)		4.84, d, (13.2)	
11	1.11, d, (6.7)	18.1, CH_3_	1.11, s	14.4, CH_3_

^a^ Recorded at ^1^H (600 MHz) and ^13^C (150 MHz); ^b^ Recorded at ^1^H (500 MHz) and ^13^C (125 MHz).

## Supplementary Materials

The following are available online at https://www.mdpi.com/1660-3397/17/3/182/s1. Figures S1–S50: HRESIMS, CD, UV, IR, 1D, and 2D NMR spectra of compounds **1**–**5**; Figures S51–S68: 1D NMR spectra of compounds **6**–**14**.

## References

[1] Kijjoa A., Sawangwong P. (2004). Drugs and cosmetics from the sea. Mar. Drugs.

[2] Bhadury P., Mohammad B.T., Wright P.C. (2006). The current status of natural products from marine fungi and their potential as anti-infective agents. J. Ind. Microbiol. Biot..

[3] Liu Y., Hu Z., Lin X., Lu C., Shen Y. (2013). A new polyketide from *Diaporthe* sp. SXZ-19, an endophytic fungal strain of *Camptotheca acuminate*. Nat. Prod. Res..

[4] Zhang C., Ondeyka J.G., Herath K.B., Guan Z., Collado J., Platas G., Pelaez F., Leavitt P.S., Gurnett A., Nare B. (2005). Tenellones A and B from a *Diaporthe* sp.: Two highly substituted benzophenone inhibitors of parasite cGMP-dependent protein kinase activity. J. Nat. Prod..

[5] Andolfi A., Boari A., Evidente M., Cimmino A., Vurro M., Ash G., Evidente A. (2015). Gulypyrones A and B and phomentrioloxins B and C produced by *Diaporthe gulyae*, a potential mycoherbicide for saffron thistle (*Carthamus lanatus*). J. Nat. Prod..

[6] Zang L.Y., Wei W., Guo Y., Wang T., Jiao R.H., Ng S.W., Tan R.X., Ge H.M. (2012). Sesquiterpenoids from the mangrove-derived endophytic fungus *Diaporthe* sp.. J. Nat. Prod..

[7] Yedukondalu N., Arora P., Wadhwa B., Malik F.A., Vishwakarma R.A., Gupta V.K., Riyazulhassan S., Ali A. (2016). Diapolic acid A-B from an endophytic fungus, *Diaporthe terebinthifolii* depicting antimicrobial and cytotoxic activity. J. Antibiot..

[8] Brady S.F., Wagenaar M.M., Singh M.P., Janso J.E., Clardy J. (2000). The cytosporones, new octaketide antibiotics isolated from an endophytic fungus. Org. Lett..

[9] Li G., Kusari S., Kusari P., Kayser O., Spiteller M. (2015). Endophytic *Diaporthe* sp. LG23 produces a potent antibacterial tetracyclic triterpenoid. J. Nat. Prod..

[10] Calcul L., Waterman C., Ma W.S., Lebar M.D., Harter C., Mutka T., Morton L., Maignan P., Van Olphen A., Kyle D.E. (2013). Screening mangrove endophytic fungi for antimalarial natural products. Mar. Drugs.

[11] Mandavid H., Rodrigues A.M., Espindola L.S., Eparvier V., Stien D. (2015). Secondary metabolites isolated from the amazonian endophytic fungus *Diaporthe* sp. SNB-GSS10. J. Nat. Prod..

[12] Schloss S., Hackl T., Herz C., Lamy E., Koch M., Rohn S., Maul R. (2017). Detection of a toxic methylated derivative of phomopsin A produced by the legume-infesting fungus *Diaporthe toxica*. J. Nat. Prod..

[13] Hu H.B., Luo Y.F., Wang P., Wang W.J., Wu J. (2018). Xanthone-derived polyketides from the thai mangrove endophytic fungus *Phomopsis* sp. xy21. Fitoterapia.

[14] Wang Y.N., Tian L., Hua H.M., Lu X., Sun S., Wu H.H., Pei Y.H. (2009). Two new compounds from the broth of the marine fungus *Penicillium griseofulvum* Y19-07. J. Asian. Nat. Prod. Res..

[15] Siddiqui S., Mahmood T., Siddiqui B.S., Faizi S. (1988). Non-terpenoidal constituents from *Azadirachta indica*. Planta Med..

[16] Devys M., Bousquet J.F., Kollmann A., Barbier M. (1980). Dihydroisocoumarins and mycophenolic acid of the culture medium of the plant pathogenic fungus *Septoria nodorum*. Phytochemistry.

[17] Avantaggiato G., Solfrizzo M., Tosi L., Zazzerini A., Fanizzi F.P., Visconti A. (1999). Isolation and characterization of phytotoxic compounds produced by *Phomopsis helianthi*. Nat. Toxins..

[18] Glauser G., Gindro K., Fringeli J., De Joffrey J.P., Rudaz S., Wolfender J.L. (2009). Differential analysis of mycoalexins in confrontation zones of grapevine fungal pathogens by ultrahigh pressure liquid chromatography/time-of-flight mass spectrometry and capillary nuclear magnetic resonance. J. Agric. Food Chem..

[19] Mali R.S., Jagtap P.G., Patil S.R., Pawar P.N. (1992). Novel AlCl_3_ catalysed syntheses of naturally occurring (±)8-hydroxy-3-rnethyl-3.4-dihydroisocoumarins. J. Chem. Soc. Chem. Comm..

[20] Xu J., Kjer J., Sendker J., Wray V., Guan H.S., Edrada R.A., Lin W.H., Wu J., Proksch P. (2009). Chromones from the endophytic fungus *Pestalotiopsis* sp. isolated from the chinese mangrove plant *Rhizophora mucronata*. J. Nat. Prod..

[21] Venkatasubbaiah P., Chilton W.S. (1992). Phytotoxins produced by *Tubakia dryina*. Mycopathologia.

[22] Krick A., Kehraus S., Gerhauser C., Klimo K., Nieger M., Maier A., Fiebig H.H., Atodiresei I., Raabe G., Fleischhauer J. (2007). Potential cancer chemopreventive in vitro activities of monomeric xanthone derivatives from the marine algicolous fungus *Monodictys putredinis*. J. Nat. Prod..

[23] Yang B., Tao H.M., Lin X.P., Wang J.F., Liao S.R., Dong J.D., Zhou X.F., Liu Y.H. (2017). Prenylated indole alkaloids and chromone derivatives from the fungus *Penicillium* sp. SCSIO041218. Tetrahedron.

[24] Skehan P., Storeng R., Scudiero D., Monks A., McMahon J., Vistica D., Warren J.T., Bokesch H., Kenney S., Boyd M.R. (1990). New colorimetric cytotoxicity assay for anticancer-drug screening. J. Natl. Cancer Inst..

[25] Frisch M.J., Trucks G.W., Schlegel H.B., Scuseria G.E., Robb M.A., Cheeseman J.R., Scalmani G., Barone V., Mennucci B., Petersson G.A. (2013). Gaussian 09, Rev. D.01.

[26] Wu P., Xue J., Yao L., Xu L., Li H., Wei X. (2015). Bisacremines E-G, three polycyclic dimeric acremines produced by acremonium persicinum SC0105. Org. Lett..

[27] Bruhn T., Schaumloffel A., Hemberger Y., Bringmann G. (2013). SpecDis: Quantifying the comparison of calculated and experimental electronic circular dichroism spectra. Chirality.

