# Supplementary material for: Chromone-Derived Polyketides from the Deep-Sea Fungus Diaporthe phaseolorum FS431
Source: Mar Drugs. 2019 Mar 20;17(3):182. doi: 10.3390/md17030182 (PMC6470668; doi:10.3390/md17030182)
Supplement: Supplementary file 1 [file marinedrugs-17-00182-s001.zip › supplementary-revised version.docx]

**Supplementary Information**

**Chromone-Derived Polyketides from the Deep-Sea Fungus *Diaporthe* *phaseolorum* FS431**

Heng Guo,^1,2^ Zhaoming Liu,^1^ Yuchan Chen,^1^ Haibo Tan,^3^ Saini Li,^1^ Haohua Li,^1^ Xiao-Xia Gao^2,*^,Hongxin Liu,^1,*^ Weimin Zhang^1,*^

^1^State Key Laboratory of Applied Microbiology Southern China, Guangdong Provincial Key Laboratory of Microbial Culture Collection and Application, Guangdong Open Laboratory of Applied Microbiology, Guangdong Institute of Microbiology, Guangzhou 510070, China

^2^College of Pharmacy, Guangdong Pharmaceutical University, Guangzhou 510006, China

^3^Program for Natural Products Chemical Biology, Key Laboratory of Plant Resources Conservation and Sustainable Utilization, Guangdong Provincial Key Laboratory of Applied Botany, South China Botanical Garden, Chinese Academy of Sciences, Guangzhou 510650, China

**Table of Contents**

[Figure S1. HRESIMS spectrum of Phaseolorin A (**1**) 5](#_Toc3912142)

[Figure S2. 1H NMR spectrum (600 MHz, CD_3_OD) of Phaseolorin A (**1**) 5](#_Toc3912143)

[Figure S3. ^13^C NMR spectrum (150 MHz, CD_3_OD) of Phaseolorin A (**1**) 6](#_Toc3912144)

[Figure S4. ^1^H-^1^H COSY spectrum (600 MHz, CD_3_OD) of Phaseolorin A (**1**) 6](#_Toc3912145)

[Figure S5. HSQC spectrum of Phaseolorin A (**1**) 7](#_Toc3912146)

[Figure S6. HMBC spectrum of Phaseolorin A (**1**) 7](#_Toc3912147)

[Figure S7. NOESY spectrum (600 MHz, CD_3_OD) of Phaseolorin A (**1**) 8](#_Toc3912148)

[Figure S8. CD spectrum of Phaseolorin A (**1**) 8](#_Toc3912149)

[Figure S9. UV spectrum of Phaseolorin A (**1**) 9](#_Toc3912150)

[Figure S10. IR spectrum of Phaseolorin A (**1**) 9](#_Toc3912151)

[Figure S11. HRESIMS spectrum of Phaseolorin B (**2**) 10](#_Toc3912152)

[Figure S12. ^1^H NMR spectrum (600 MHz, CD_3_COCD_3_) of Phaseolorin B (**2**) 10](#_Toc3912153)

[Figure S13. ^13^C NMR spectrum (150 MHz, CD_3_COCD_3_) of Phaseolorin B (**2**) 11](#_Toc3912154)

[Figure S14. ^1^H-^1^H COSY spectrum (600 MHz, CD_3_COCD_3_) of Phaseolorin B (**2**) 11](#_Toc3912155)

[Figure S15. HSQC spectrum of Phaseolorin B (**2**) 12](#_Toc3912156)

[Figure S16. HMBC spectrum of Phaseolorin B (**2**) 12](#_Toc3912157)

[Figure S17. NOESY spectrum (600 MHz, CD_3_COCD_3_) of Phaseolorin B (**2**) 13](#_Toc3912158)

[Figure S18. CD spectrum of Phaseolorin B (**2**) 13](#_Toc3912159)

[Figure S19. UV spectrum of Phaseolorin B (**2**) 14](#_Toc3912160)

[Figure S20. IR spectrum of Phaseolorin B (**2**) 14](#_Toc3912161)

[Figure S21. HRESIMS spectrum of Phaseolorin C (**3**) 15](#_Toc3912162)

[Figure S22. ^1^H NMR spectrum (600 MHz, CD_3_COCD_3_) of Phaseolorin C (**3**) 15](#_Toc3912163)

[Figure S23. ^13^C NMR spectrum (150 MHz, CD_3_COCD_3_) of Phaseolorin C (**3**) 16](#_Toc3912164)

[Figure S24. ^1^H-^1^H COSY spectrum (600 MHz, CD_3_COCD_3_) of Phaseolorin C (**3**) 16](#_Toc3912165)

[Figure S25. HSQC spectrum of Phaseolorin C (**3**) 17](#_Toc3912166)

[Figure S26. HMBC spectrum of Phaseolorin C (**3**) 17](#_Toc3912167)

[Figure S27. NOESY spectrum (600 MHz, CD_3_COCD_3_) of Phaseolorin C (**3**) 18](#_Toc3912168)

[Figure S28. CD spectrum of Phaseolorin C (**3**) 18](#_Toc3912169)

[Figure S29. UV spectrum of Phaseolorin C (**3**) 19](#_Toc3912170)

[Figure S30. IR spectrum of Phaseolorin C (**3**) 19](#_Toc3912171)

[Figure S31. HRESIMS spectrum of Phaseolorin D (**4**) 20](#_Toc3912172)

[Figure S32. ^1^H NMR spectrum (500 MHz, CD_3_OD) of Phaseolorin D (**4**) 20](#_Toc3912173)

[Figure S33. ^13^C NMR spectrum (150 MHz, CD_3_OD) of Phaseolorin D (**4**) 21](#_Toc3912174)

[Figure S34. ^1^H-^1^H COSY spectrum (500 MHz, CD_3_OD) of Phaseolorin D (**4**) 21](#_Toc3912175)

[Figure S35. HSQC spectrum of Phaseolorin D (**4**) 22](#_Toc3912176)

[Figure S36. HMBC spectrum of Phaseolorin D (**4**) 22](#_Toc3912177)

[Figure S37. NOESY spectrum (600 MHz, CD_3_OD) of Phaseolorin D (**4**) 23](#_Toc3912178)

[Figure S38. CD spectrum of Phaseolorin D (**4**) 23](#_Toc3912179)

[Figure S39. UV spectrum of Phaseolorin D (**4**) 24](#_Toc3912180)

[Figure S40. IR spectrum of Phaseolorin D (**4**) 24](#_Toc3912181)

[Figure S41. HRESIMS spectrum of Phaseolorin E (**5**) 25](#_Toc3912182)

[Figure S42. ^1^H NMR spectrum (600 MHz, CD_3_OD) of Phaseolorin E (**5**) 25](#_Toc3912183)

[Figure S43. ^13^C NMR spectrum (150 MHz, CD_3_OD) of Phaseolorin E (**5**) 26](#_Toc3912184)

[Figure S44. ^1^H-^1^H COSY spectrum (600 MHz, CD_3_OD) of Phaseolorin E (**5**) 26](#_Toc3912185)

[Figure S45. HSQC spectrum of Phaseolorin E (**5**) 27](#_Toc3912186)

[Figure S46. HMBC spectrum of Phaseolorin E (**5**) 27](#_Toc3912187)

[Figure S47. NOESY spectrum (600 MHz, CD_3_COCD_3_) of Phaseolorin E (**5**) 28](#_Toc3912188)

[Figure S48. CD spectrum of Phaseolorin E (**5**) 28](#_Toc3912189)

[Figure S49. UV spectrum of Phaseolorin E (**5**) 29](#_Toc3912190)

[Figure S50. IR spectrum of Phaseolorin E (**5**) 29](#_Toc3912191)

[Figure S51. ^1^H NMR spectrum (600 MHz, CD_3_OD) of Phomoxanthone G (**6)** 30](#_Toc3912192)

[Figure S52. ^13^C NMR spectrum (150 MHz, CD_3_OD) of Phomoxanthone G (**6)** 30](#_Toc3912193)

[Figure S53. ^1^H NMR spectrum (600 MHz, CD_3_OD) of Compound **7** 31](#_Toc3912194)

[Figure S54. ^13^C NMR spectrum (150 MHz, CD_3_OD) of Compound **7** 31](#_Toc3912195)

[Figure S55. ^1^H NMR spectrum (600 MHz, CD_3_OD) of Compound **8** 32](#_Toc3912196)

[Figure S56. ^13^C NMR spectrum (150 MHz, CD_3_OD) of Compound **8** 32](#_Toc3912197)

[Figure S57. ^1^H NMR spectrum (600 MHz, C_5_D_5_N) of Compound **9** 33](#_Toc3912198)

[Figure S58. ^13^C NMR spectrum (150 MHz, C_5_D_5_N) of Compound **9** 33](#_Toc3912199)

[Figure S59. ^1^H NMR spectrum (500 MHz, CD_3_OD) of Compound **10** 34](#_Toc3912200)

[Figure S60. ^13^C NMR spectrum (125 MHz, CD_3_OD) of Compound **10** 34](#_Toc3912201)

[Figure S61. ^1^H NMR spectrum (500 MHz, CD_3_OD) of Compound **11** 35](#_Toc3912202)

[Figure S62. ^13^C NMR spectrum (125 MHz, CD_3_OD) of Compound **11** 35](#_Toc3912203)

[Figure S63. ^1^H NMR spectrum (600 MHz, CD_3_OD) of Compound **12** 36](#_Toc3912204)

[Figure S64. ^13^C NMR spectrum (150 MHz, CD_3_OD) of Compound **12** 36](#_Toc3912205)

[Figure S65. ^1^H NMR spectrum (500 MHz, CD_3_OD) of Compound **13** 37](#_Toc3912206)

[Figure S66. ^13^C NMR spectrum (125 MHz, CD_3_OD) of Compound **13** 37](#_Toc3912207)

[Figure S67. ^1^H NMR spectrum (600 MHz, DMSO-*d*_6_) of Compound **14** 38](#_Toc3912208)

[Figure S68. ^13^C NMR spectrum (150 MHz, DMSO-*d*_6_) of Compound **14** 38](#_Toc3912209)

[Figure S69. Fragment ions of compound **1** from the crude extract of FS431 (Retention time: 11.0 min) 39](#_Toc3912210)

[Table S1**.** Energy analysis for the Conformers of Phaseolorin A (**1**). 39](#_Toc3912211)

[Figure S70. B3LYP/6-31G(d) optimized low-energy conformers of Phaseolorin A (**1**) 40](#_Toc3912212)

[Table S2. Energy analysis for the Conformers of Phaseolorin C (**3**). 40](#_Toc3912213)

[Figure S71. B3LYP/6-31G(d) optimized low-energy conformers of Phaseolorin C (**3**) 41](#_Toc3912214)


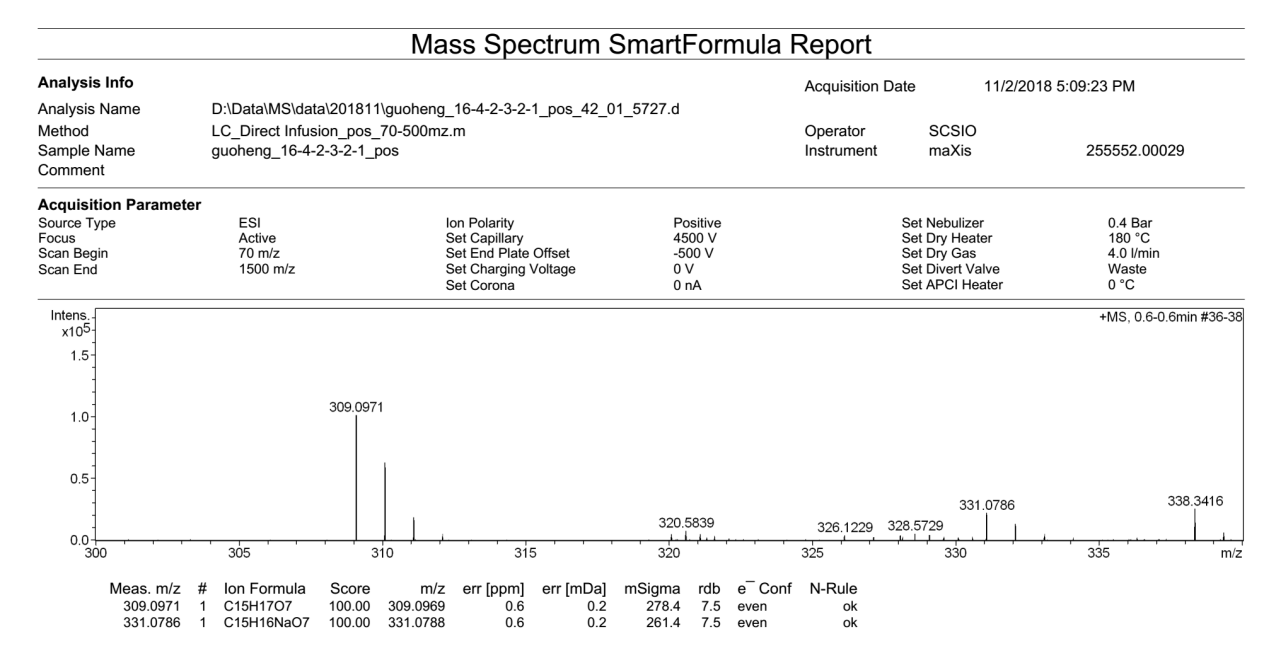


Figure S1. HRESIMS spectrum of Phaseolorin A (**1**)

Figure S2. 1H NMR spectrum (600 MHz, CD_3_OD) of Phaseolorin A (**1**)

Figure S3. ^13^C NMR spectrum (150 MHz, CD_3_OD) of Phaseolorin A (**1**)

Figure S4. ^1^H-^1^H COSY spectrum (600 MHz, CD_3_OD) of Phaseolorin A (**1**)

Figure S5. HSQC spectrum of Phaseolorin A (**1**)

Figure S6. HMBC spectrum of Phaseolorin A (**1**)

Figure S7. NOESY spectrum (600 MHz, CD_3_OD) of Phaseolorin A (**1**)


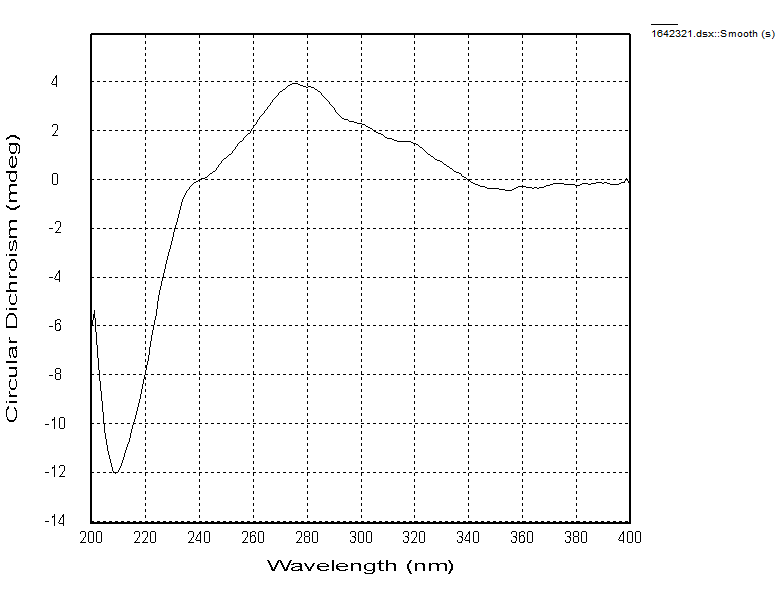


Figure S8. CD spectrum of Phaseolorin A (**1**)


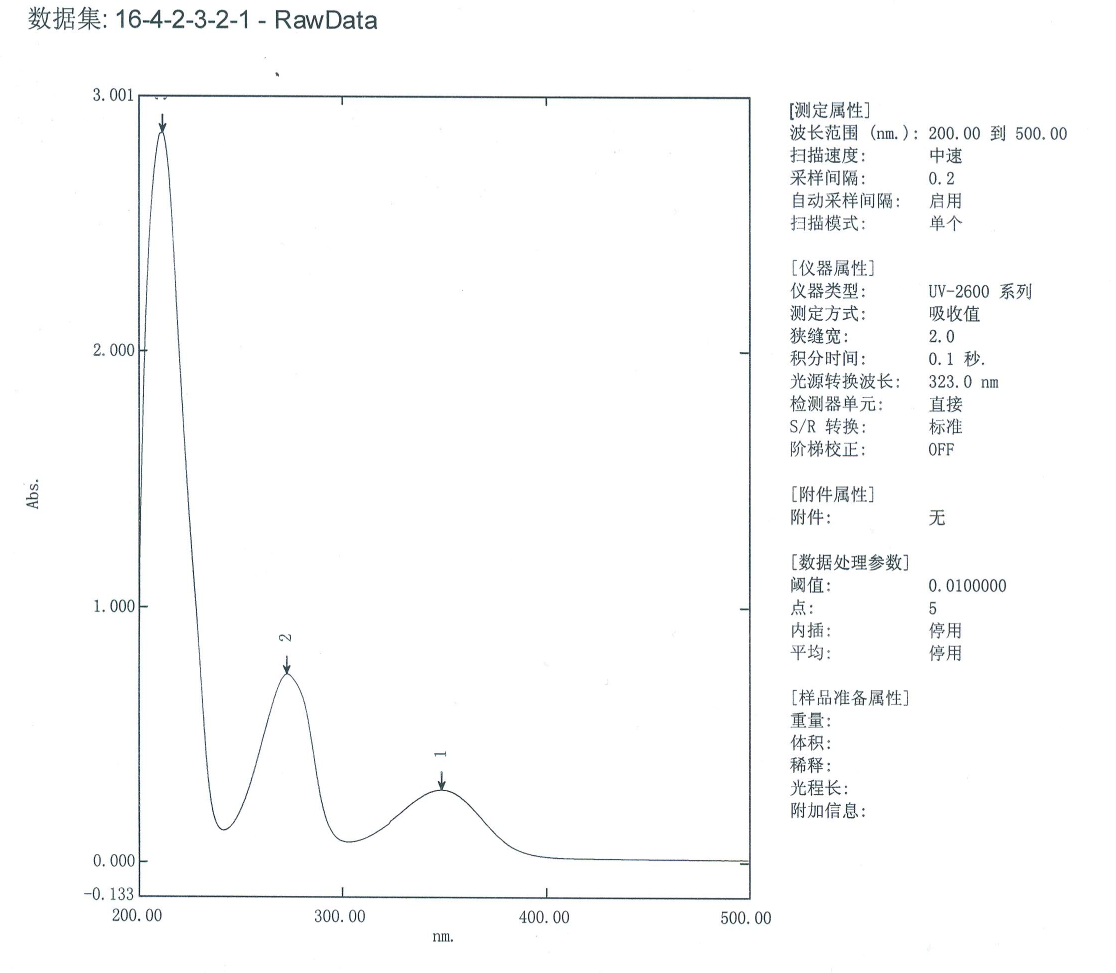


Figure S9. UV spectrum of Phaseolorin A (**1**)


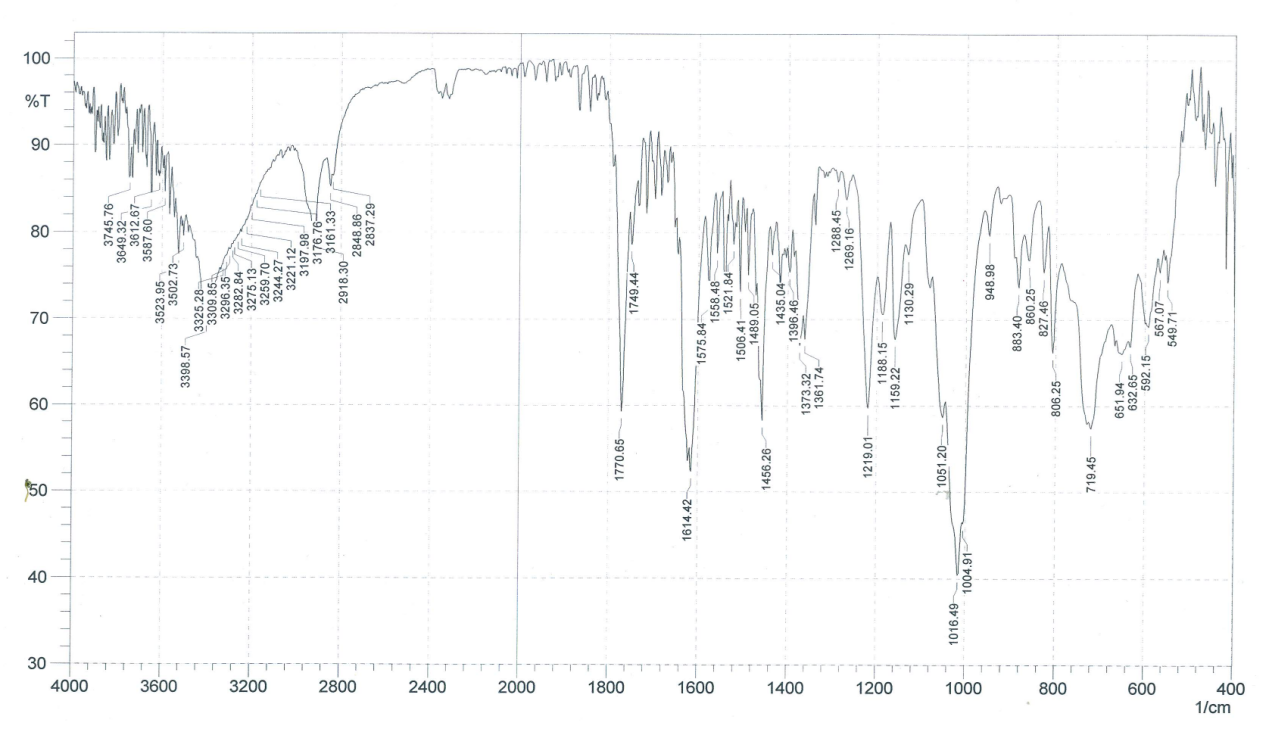


Figure S10. IR spectrum of Phaseolorin A (**1**)


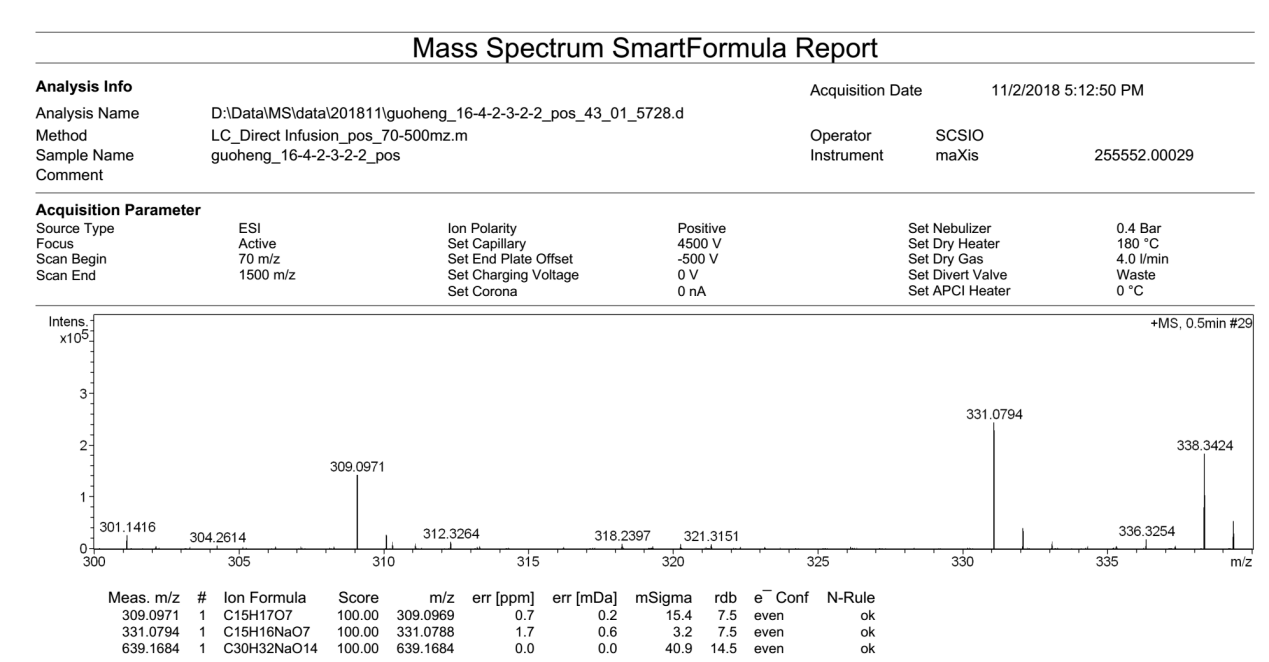


Figure S11. HRESIMS spectrum of Phaseolorin B (**2**)

Figure S12. ^1^H NMR spectrum (600 MHz, CD_3_COCD_3_) of Phaseolorin B (**2**)

Figure S13. ^13^C NMR spectrum (150 MHz, CD_3_COCD_3_) of Phaseolorin B (**2**)

Figure S14. ^1^H-^1^H COSY spectrum (600 MHz, CD_3_COCD_3_) of Phaseolorin B (**2**)

Figure S15. HSQC spectrum of Phaseolorin B (**2**)

Figure S16. HMBC spectrum of Phaseolorin B (**2**)

Figure S17. NOESY spectrum (600 MHz, CD_3_COCD_3_) of Phaseolorin B (**2**)


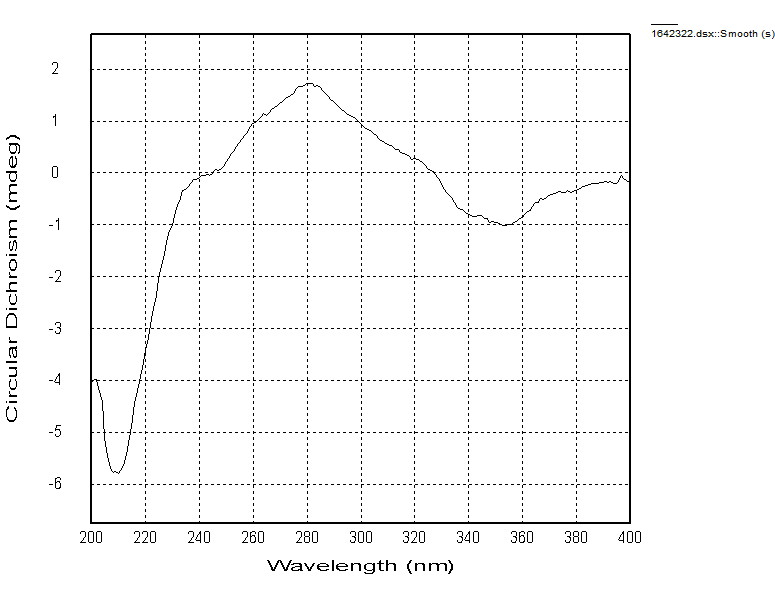


Figure S18. CD spectrum of Phaseolorin B (**2**)


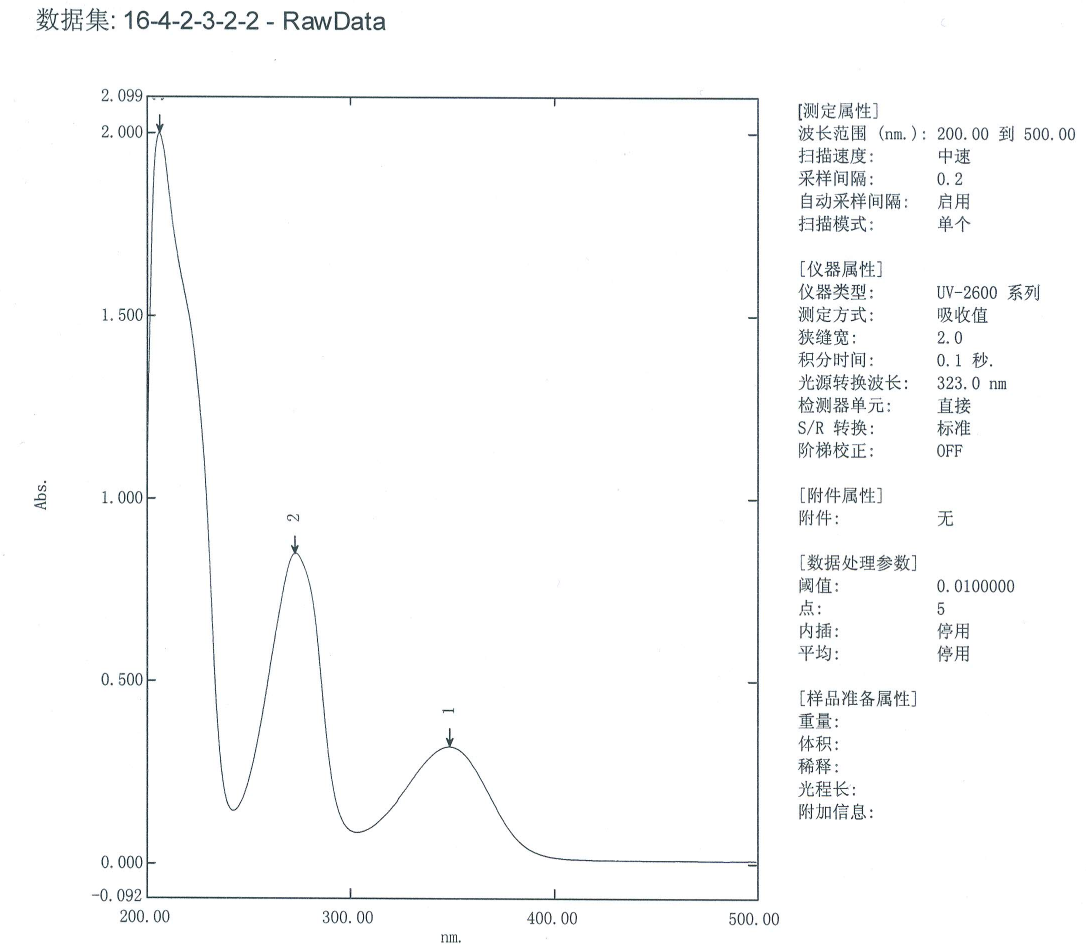


Figure S19. UV spectrum of Phaseolorin B (**2**)


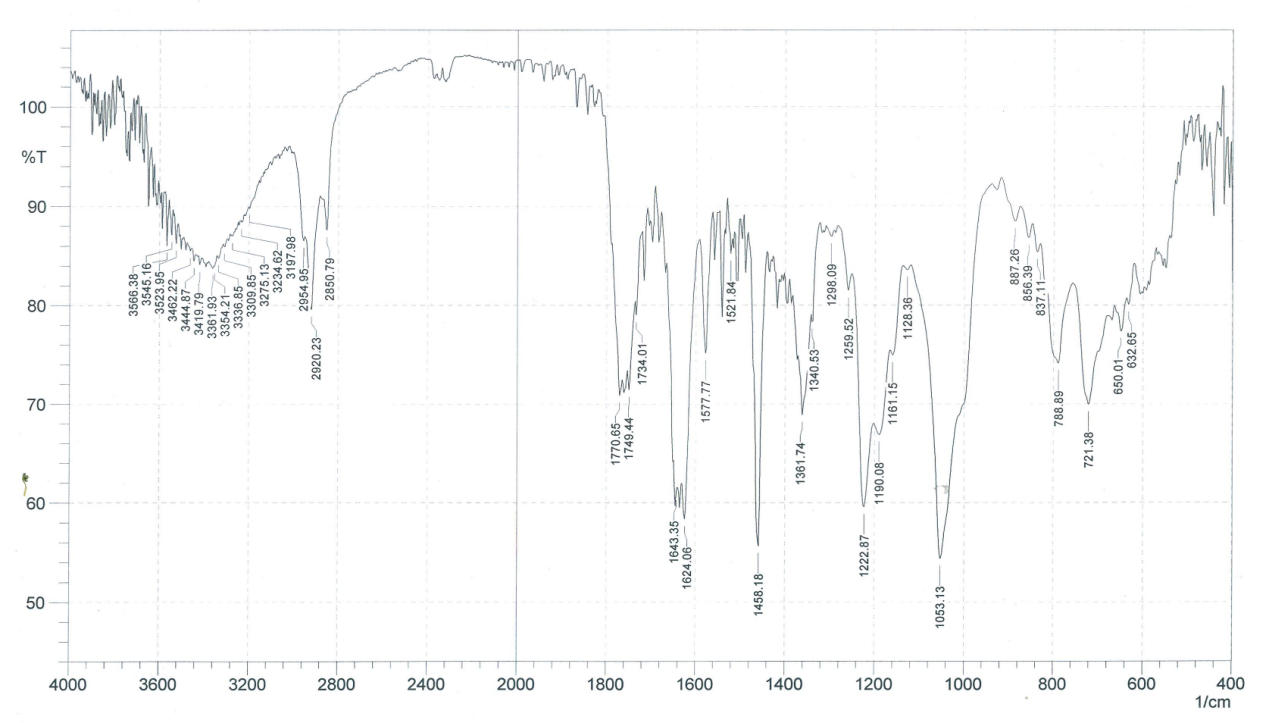


Figure S20. IR spectrum of Phaseolorin B (**2**)


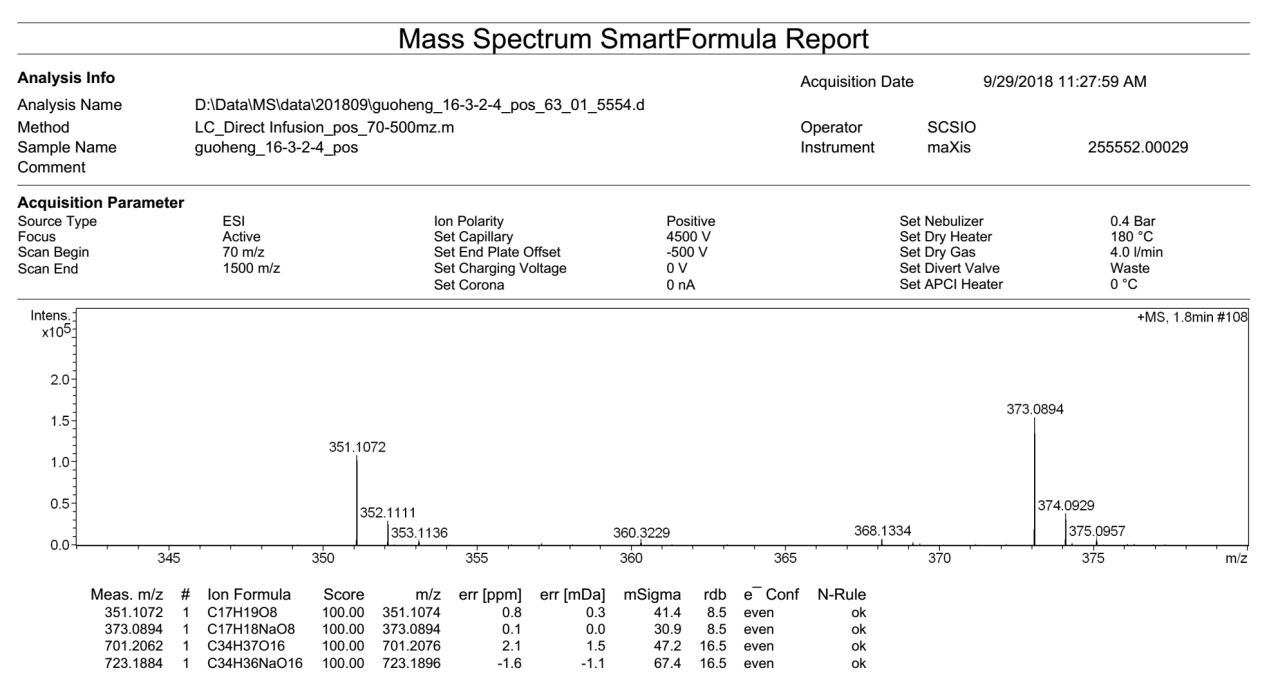


Figure S21. HRESIMS spectrum of Phaseolorin C (**3**)

Figure S22. ^1^H NMR spectrum (600 MHz, CD_3_COCD_3_) of Phaseolorin C (**3**)

Figure S23. ^13^C NMR spectrum (150 MHz, CD_3_COCD_3_) of Phaseolorin C (**3**)

Figure S24. ^1^H-^1^H COSY spectrum (600 MHz, CD_3_COCD_3_) of Phaseolorin C (**3**)

Figure S25. HSQC spectrum of Phaseolorin C (**3**)

Figure S26. HMBC spectrum of Phaseolorin C (**3**)

Figure S27. NOESY spectrum (600 MHz, CD_3_COCD_3_) of Phaseolorin C (**3**)


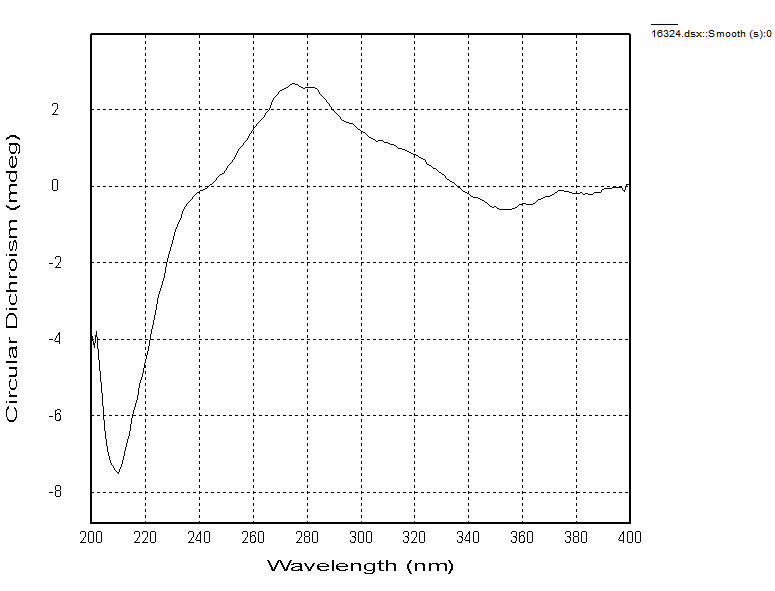


Figure S28. CD spectrum of Phaseolorin C (**3**)


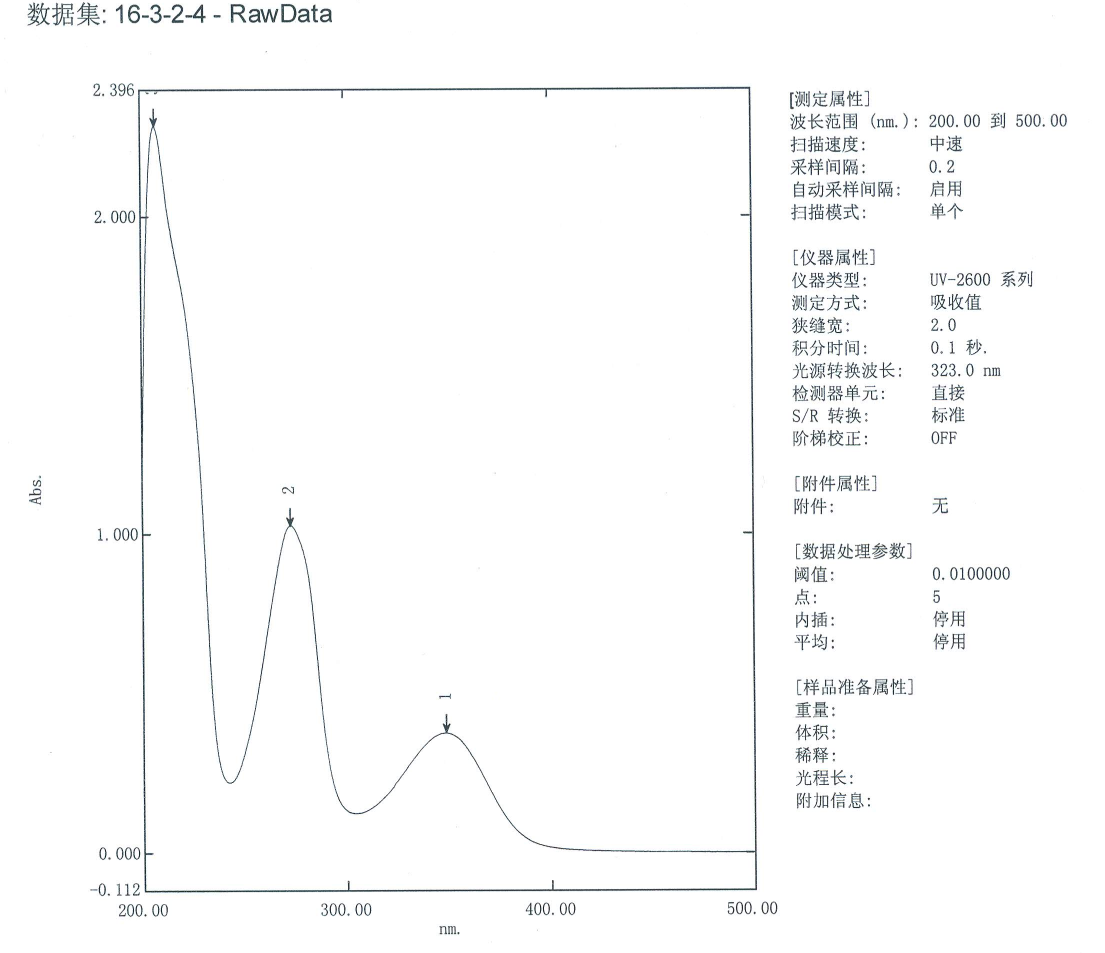


Figure S29. UV spectrum of Phaseolorin C (**3**)


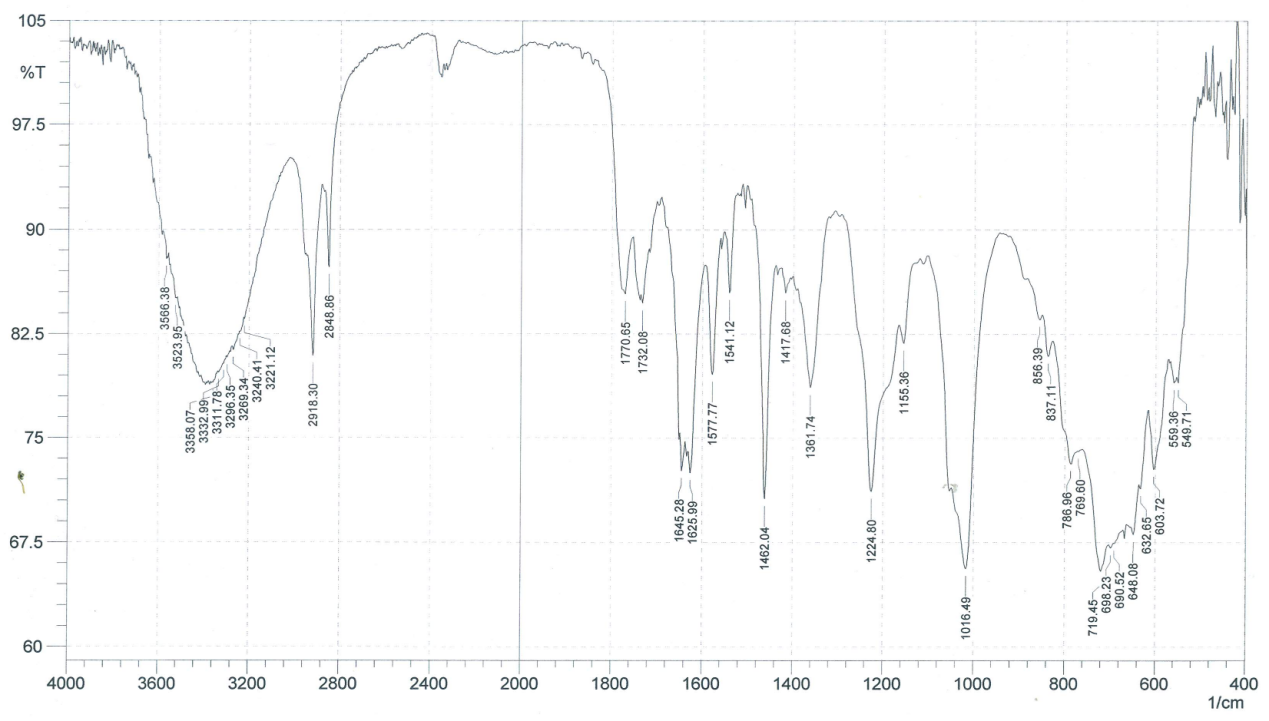


Figure S30. IR spectrum of Phaseolorin C (**3**)


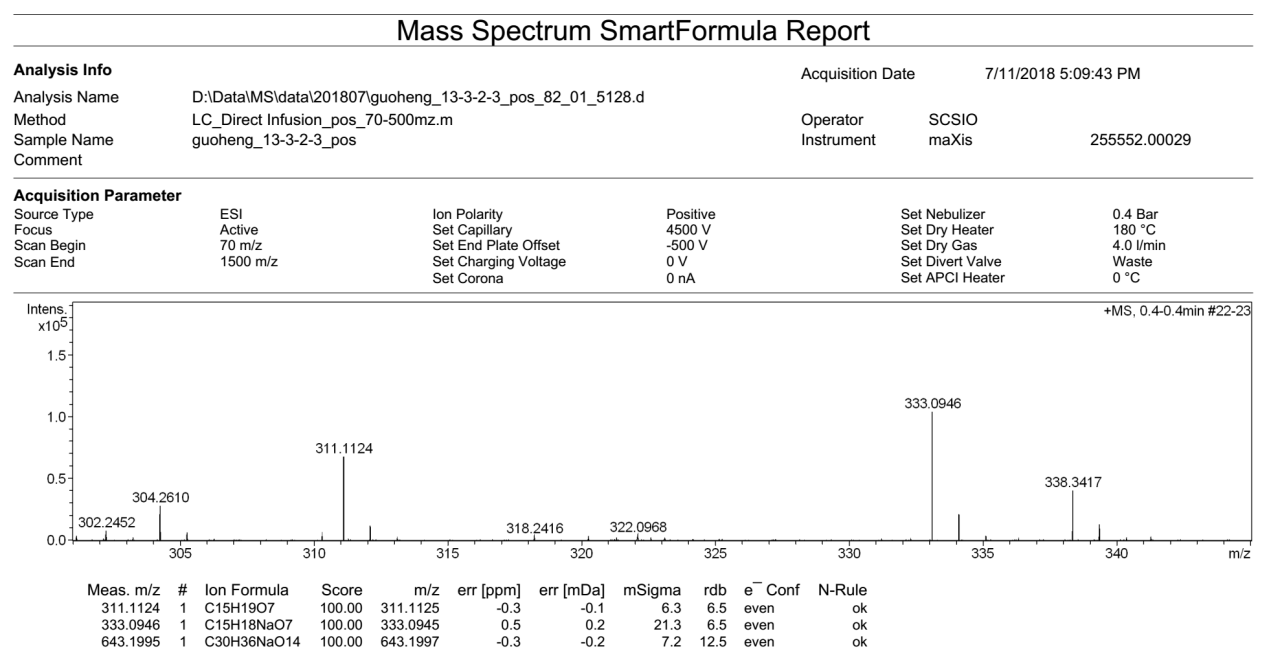


Figure S31. HRESIMS spectrum of Phaseolorin D (**4**)

Figure S32. ^1^H NMR spectrum (500 MHz, CD_3_OD) of Phaseolorin D (**4**)

Figure S33. ^13^C NMR spectrum (150 MHz, CD_3_OD) of Phaseolorin D (**4**)

Figure S34. ^1^H-^1^H COSY spectrum (500 MHz, CD_3_OD) of Phaseolorin D (**4**)

Figure S35. HSQC spectrum of Phaseolorin D (**4**)

Figure S36. HMBC spectrum of Phaseolorin D (**4**)

Figure S37. NOESY spectrum (600 MHz, CD_3_OD) of Phaseolorin D (**4**)


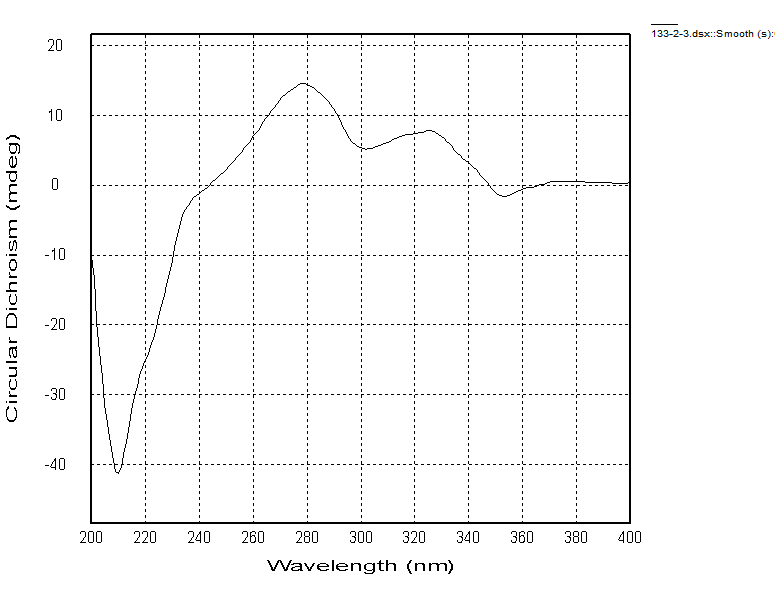


Figure S38. CD spectrum of Phaseolorin D (**4**)


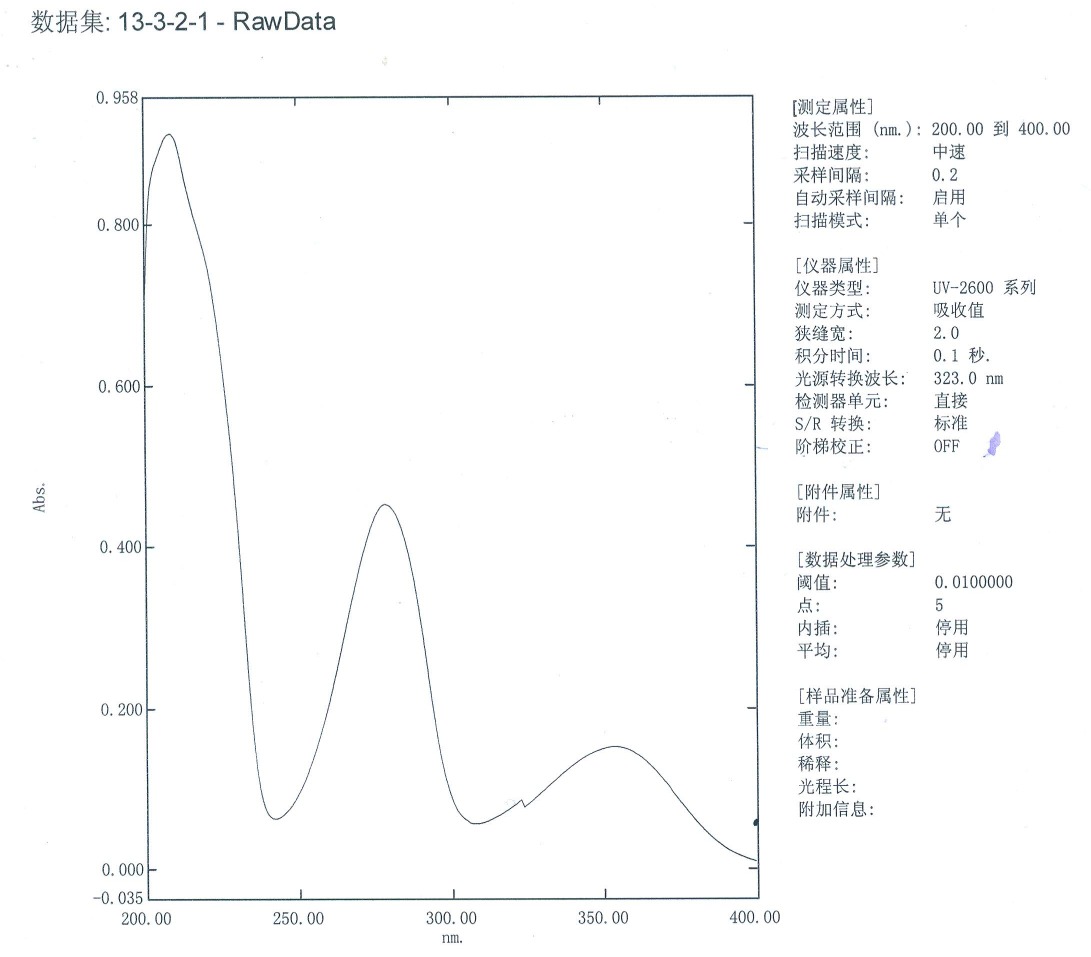


Figure S39. UV spectrum of Phaseolorin D (**4**)


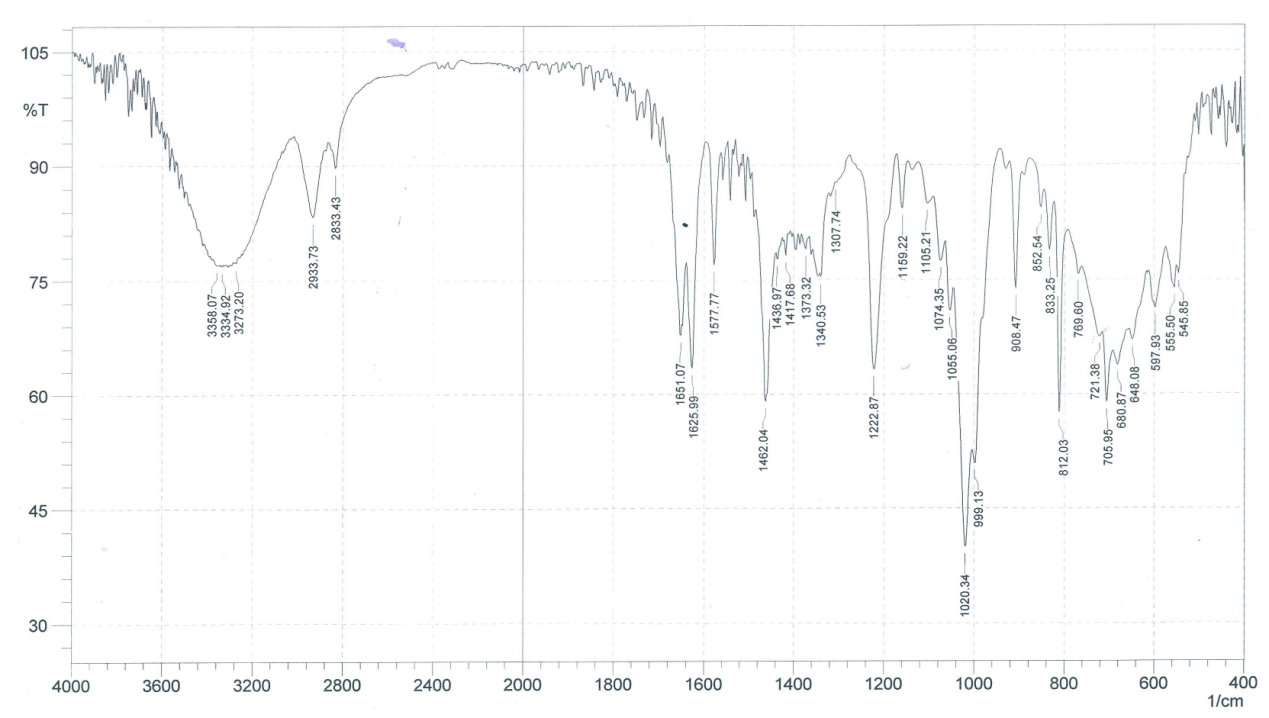


Figure S40. IR spectrum of Phaseolorin D (**4**)


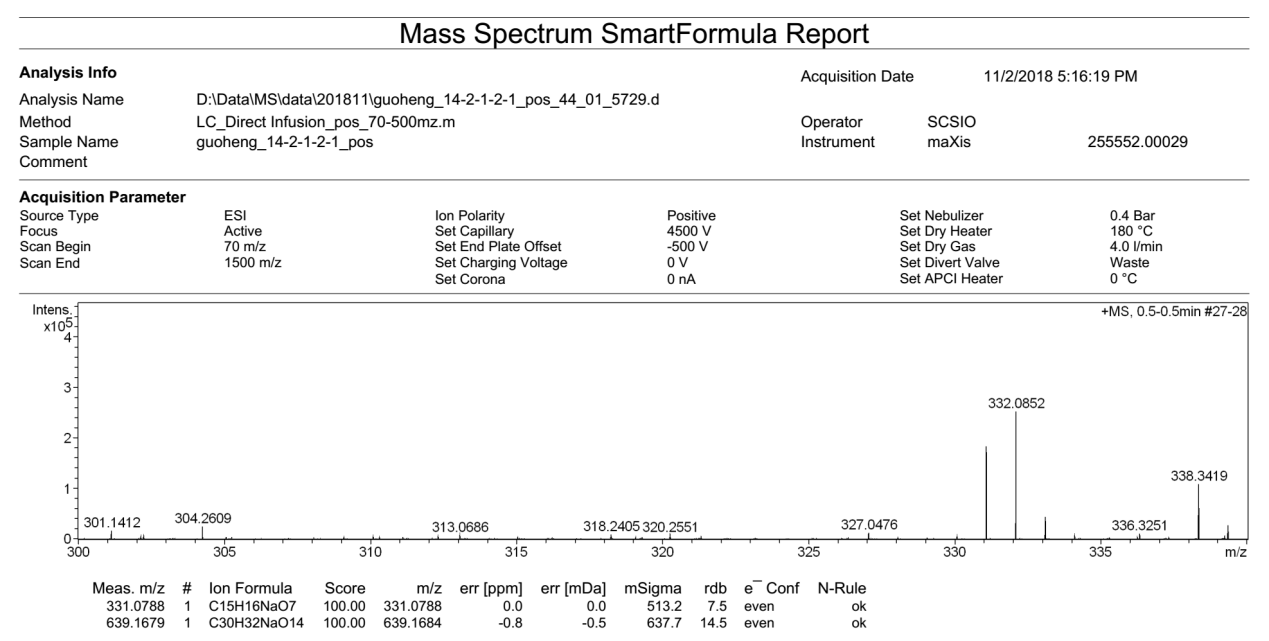


Figure S41. HRESIMS spectrum of Phaseolorin E (**5**)

Figure S42. ^1^H NMR spectrum (600 MHz, CD_3_OD) of Phaseolorin E (**5**)

Figure S43. ^13^C NMR spectrum (150 MHz, CD_3_OD) of Phaseolorin E (**5**)

Figure S44. ^1^H-^1^H COSY spectrum (600 MHz, CD_3_OD) of Phaseolorin E (**5**)

Figure S45. HSQC spectrum of Phaseolorin E (**5**)

Figure S46. HMBC spectrum of Phaseolorin E (**5**)

Figure S47. NOESY spectrum (600 MHz, CD_3_COCD_3_) of Phaseolorin E (**5**)


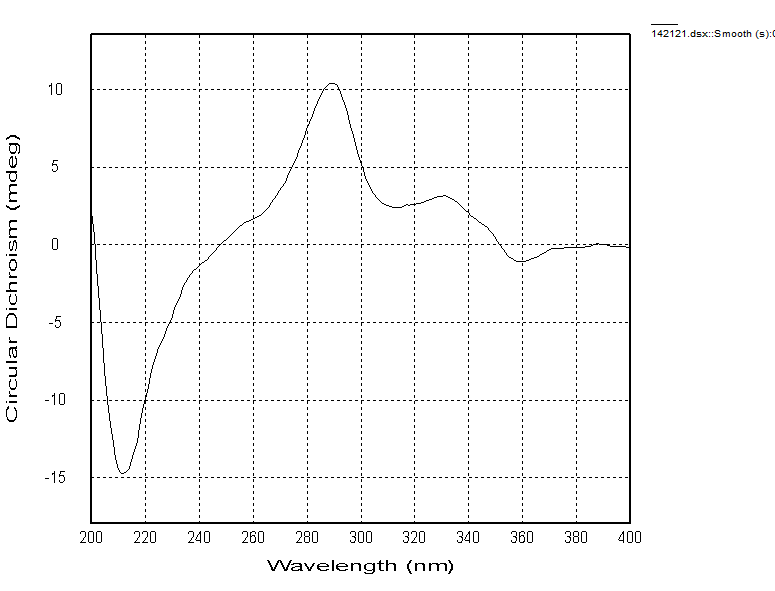


Figure S48. CD spectrum of Phaseolorin E (**5**)


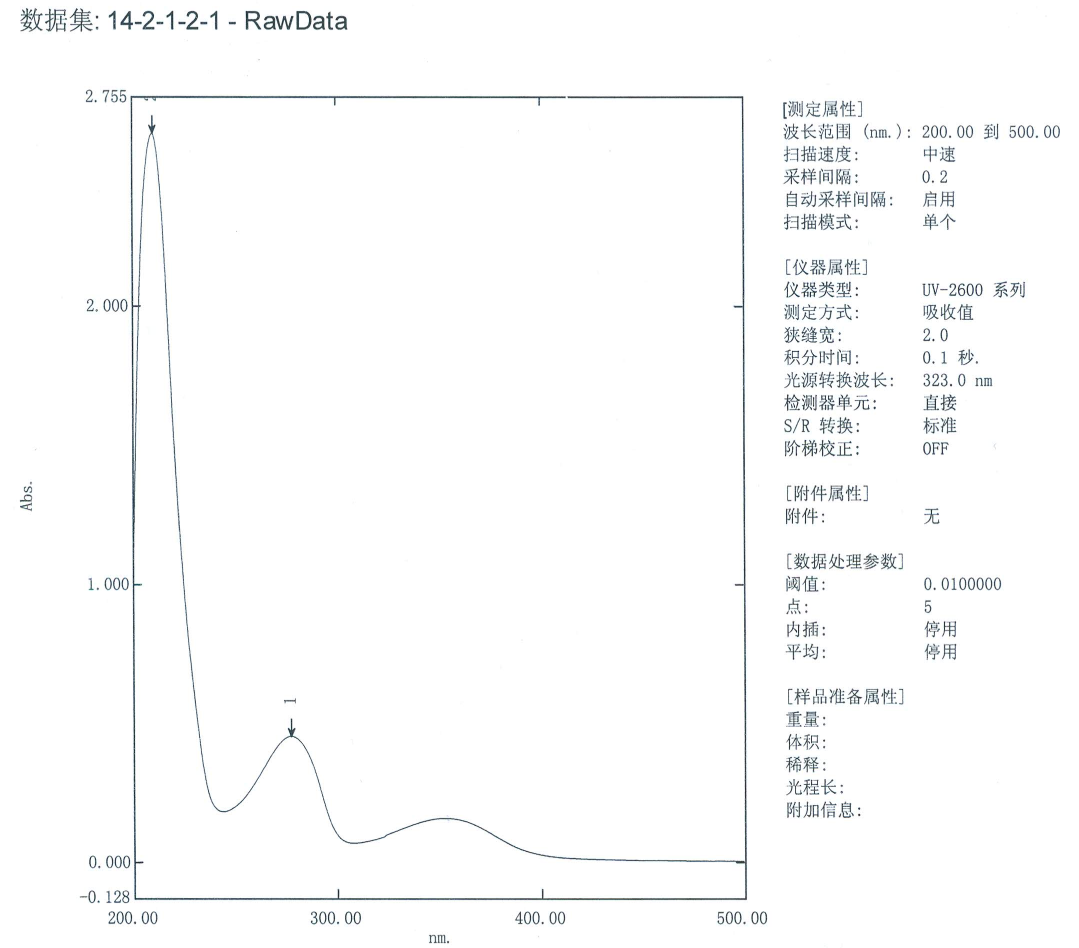


Figure S49. UV spectrum of Phaseolorin E (**5**)


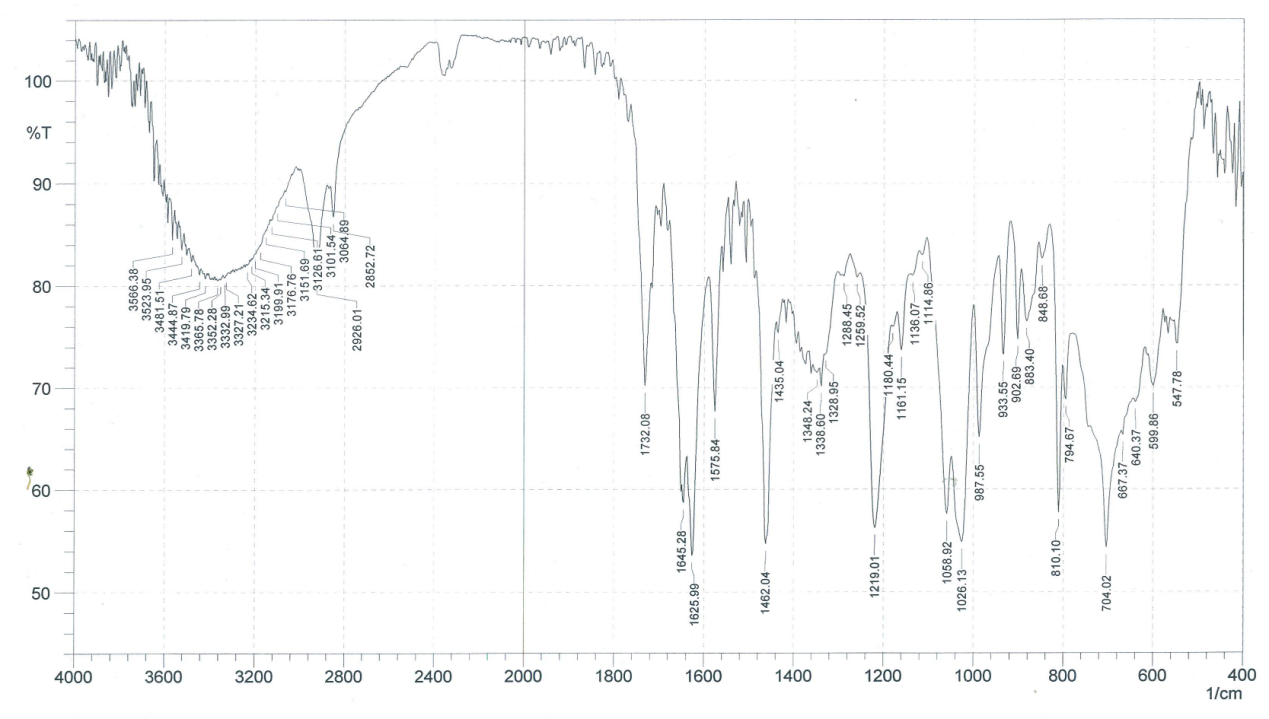


Figure S50. IR spectrum of Phaseolorin E (**5**)

Figure S51. ^1^H NMR spectrum (600 MHz, CD_3_OD) of Phomoxanthone G (**6)**

Figure S52. ^13^C NMR spectrum (150 MHz, CD_3_OD) of Phomoxanthone G (**6)**

Figure S53. ^1^H NMR spectrum (600 MHz, CD_3_OD) of Compound **7**

Figure S54. ^13^C NMR spectrum (150 MHz, CD_3_OD) of Compound **7**

Figure S55. ^1^H NMR spectrum (600 MHz, CD_3_OD) of Compound **8**

Figure S56. ^13^C NMR spectrum (150 MHz, CD_3_OD) of Compound **8**

Figure S57. ^1^H NMR spectrum (600 MHz, C_5_D_5_N) of Compound **9**

Figure S58. ^13^C NMR spectrum (150 MHz, C_5_D_5_N) of Compound **9**

Figure S59. ^1^H NMR spectrum (500 MHz, CD_3_OD) of Compound **10**

Figure S60. ^13^C NMR spectrum (125 MHz, CD_3_OD) of Compound **10**

Figure S61. ^1^H NMR spectrum (500 MHz, CD_3_OD) of Compound **11**

Figure S62. ^13^C NMR spectrum (125 MHz, CD_3_OD) of Compound **11**

Figure S63. ^1^H NMR spectrum (600 MHz, CD_3_OD) of Compound **12**

Figure S64. ^13^C NMR spectrum (150 MHz, CD_3_OD) of Compound **12**

Figure S65. ^1^H NMR spectrum (500 MHz, CD_3_OD) of Compound **13**

Figure S66. ^13^C NMR spectrum (125 MHz, CD_3_OD) of Compound **13**

Figure S67. ^1^H NMR spectrum (600 MHz, DMSO-*d*_6_) of Compound **14**

Figure S68. ^13^C NMR spectrum (150 MHz, DMSO-*d*_6_) of Compound **14**


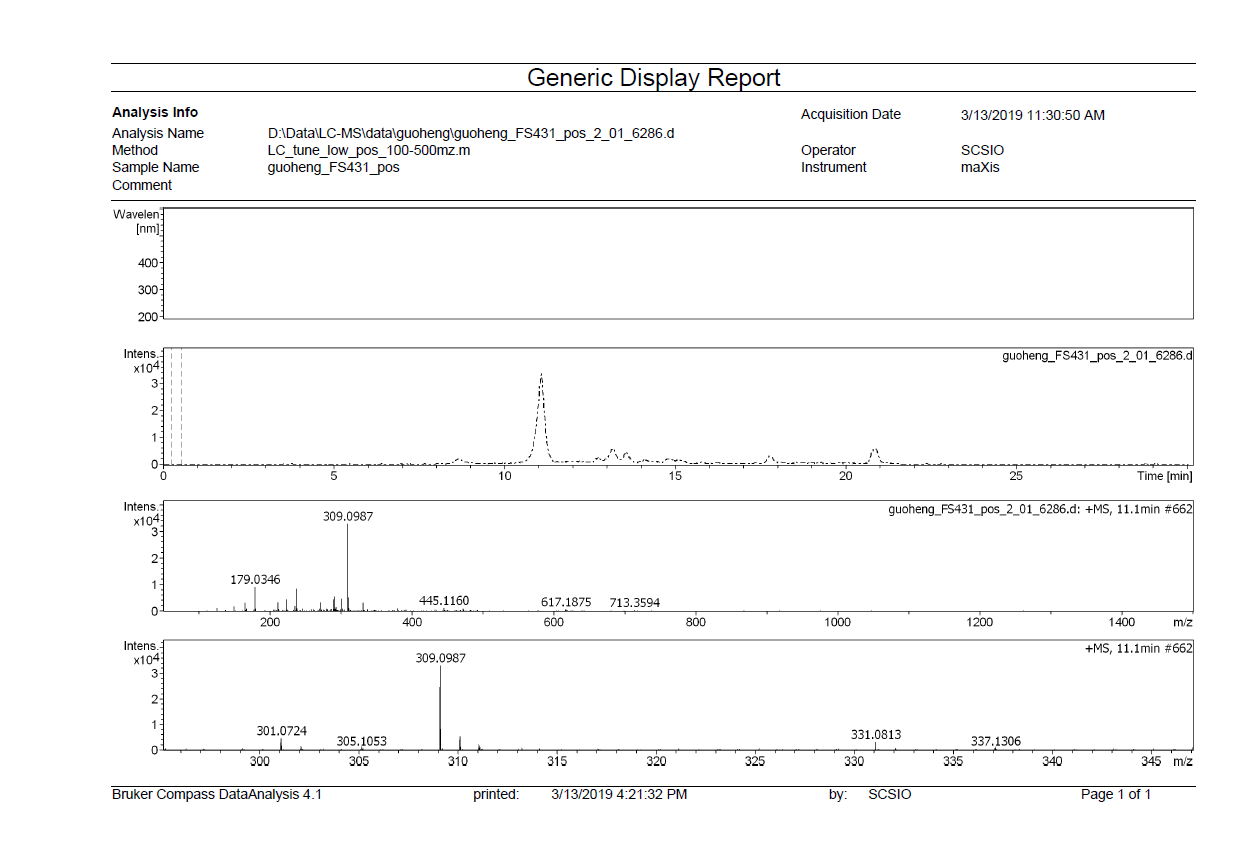


Figure S69. Fragment ions of compound **1** from the crude extract of FS431 (Retention time: 11.0 min)

Table S1. Energy analysis for the Conformers of Phaseolorin A (**1**).

| compounds | Conformation | G (Hartree) | G (Kcal/mol) | ∆G (Kcal/mol) | Boltzmann Dist (%) |
| --- | --- | --- | --- | --- | --- |
| **1** | **1-**a | -1107.89651520 | -695208.2762 | 0.503376551 | 17.03% |
|  | **1-**b | -1107.89731739 | -695208.7796 | 0 | 39.85% |
|  | **1-**c | -1107.89657995 | -695208.3168 | 0.462745739 | 18.24% |
|  | **1-**d | -1107.89638182 | -695208.1925 | 0.587072888 | 14.79% |
|  | **1-**e | -1107.89602146 | -695207.9664 | 0.813199833 | 10.09% |


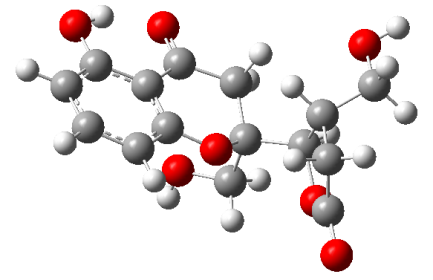

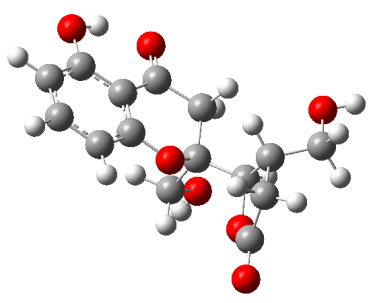

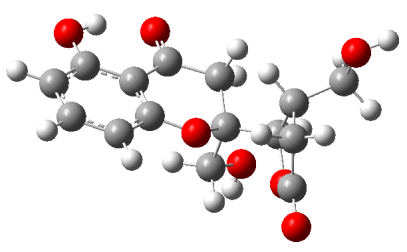


**1-**a **1-**b **1-**c


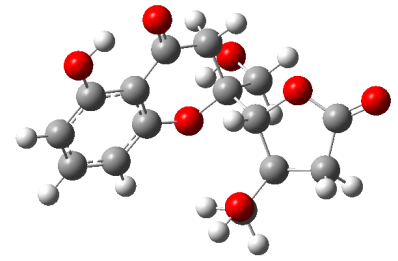

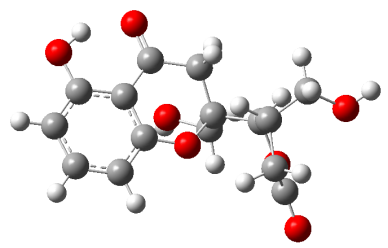


**1-**d **1-**e

Figure S70. B3LYP/6-31G(d) optimized low-energy conformers of Phaseolorin A (**1**)

Table S2. Energy analysis for the Conformers of Phaseolorin C (**3**).

| compounds | Conformation | G (Hartree) | G (Kcal/mol) | ∆G (Kcal/mol) | Boltzmann Dist (%) |
| --- | --- | --- | --- | --- | --- |
| **3** | **3-**a | -1260.56974165 | -791011.1685 | 0 | 32.60% |
|  | **3-**b | -1260.56836463 | -791010.3045 | 0.864084043 | 7.58% |
|  | **3-**c | -1260.56916972 | -791010.8096 | 0.358887734 | 17.78% |
|  | **3-**d | -1260.56908753 | -791010.7581 | 0.410462197 | 16.30% |
|  | **3-**e | -1260.56930498 | -791010.8945 | 0.274011691 | 20.52% |
|  | **3-**f | -1260.56801137 | -791010.0828 | 1.085755718 | 5.21% |


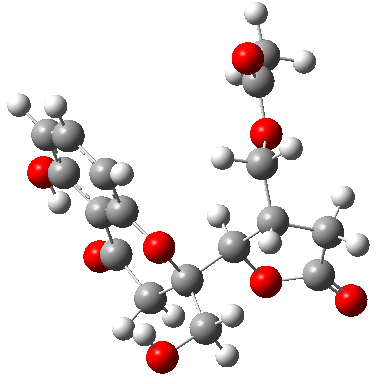

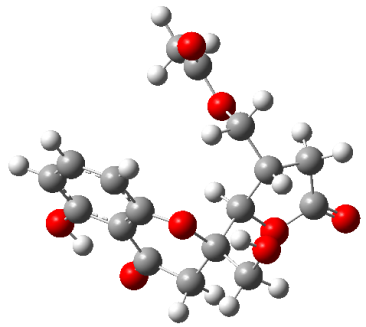

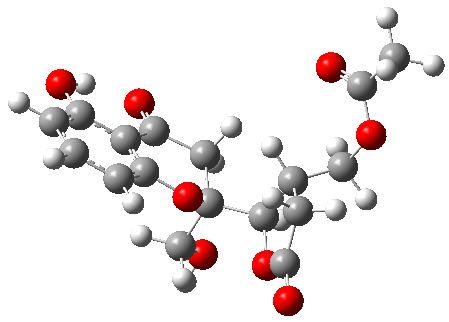


**3-**a **3-**b **3-**c


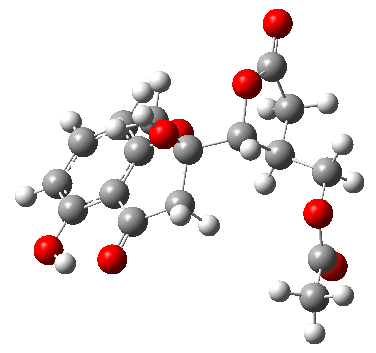

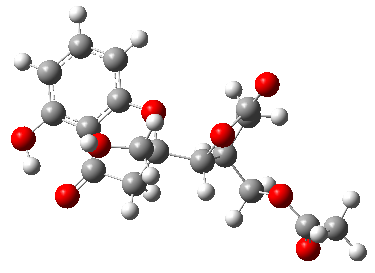

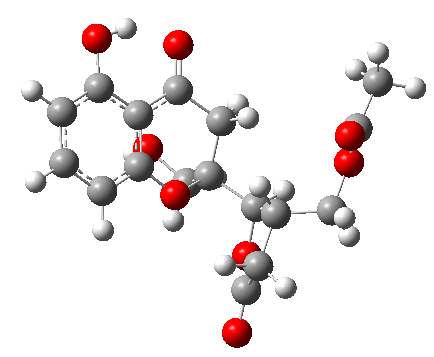


**3-**d **3-**e **3-**f

Figure S71. B3LYP/6-31G(d) optimized low-energy conformers of Phaseolorin C (**3**)
